# HPLC/ESI-MS and NMR Analysis of Chemical Constitutes in Bioactive Extract from the Root Nodule of *Vaccinium emarginatum*

**DOI:** 10.3390/ph14111098

**Published:** 2021-10-28

**Authors:** Hsiang-Ming Huang, Chien-Yi Ho, Geng-Ruei Chang, Wei-Yau Shia, Cheng-Hung Lai, Chih-Hao Chao, Chao-Min Wang

**Affiliations:** 1 Neurosurgery Department, China Medical University Hsinchu Hospital, Hsinchu 302, Taiwan; d10190168@gmail.com; 2 Department of Biomedical Imaging and Radiological Science, China Medical University, Taichung 404, Taiwan; samsam172@yahoo.com.tw; 3 Division of Family Medicine, Physical Examination Center, and Department of Medical Research, China Medical University Hsinchu Hospital, Hsinchu 302, Taiwan; 4 Department of Veterinary Medicine, National Chiayi University, 580 Xinmin Road, Chiayi 600, Taiwan; grchang@mail.ncyu.edu.tw; 5 Department of Veterinary Medicine, National Chung-Hsing University, 145 XingDa Road, Taichung 402, Taiwan; vmwyshia@nchu.edu.tw (W.-Y.S.); chlai@dragon.nchu.edu.tw (C.-H.L.); 6 Division of Chest Medicine, Attending Physician of Chang Bing Show Chwan Memorial Hospital, 6 Lugong Road, Lukang Township, Changhua 505, Taiwan

**Keywords:** *Vaccinium emarginatum*, anti-cancer, anti-bacterial, procyanidins

## Abstract

*Vaccinium emarginatum* Hayata is a medicinal plant that has been historically used in ethnopharmacy to treat diseases in Taiwan. The objective of this study is to evaluate the anti-cancer and anti-bacterial constitutes from the root nodule extract of *V. emarginatum*. The chemical composition of *V. emarginatum* fractions was analyzed by high-performance liquid chromatography-electrospray ionization tandem mass spectrometry (HPLC-ESI-MS/MS) and the chemical constitutes were isolated and structurally identified by nuclear magnetic resonance (NMR) spectroscopy. Bioassay-guided chromatography showed that the ethyl acetate (EA) fraction was bioactive on the hepatocellular carcinoma (HepG2). By LC-ESI-MS/MS analysis, twenty peaks of EA fraction were partially identified and the phytochemical investigation of the fractions led to the isolation and identification of protocatuchuic acid (**1**), epicatechin (**2**), catechin (**3**), procyanidin B3 (**4**), procyanidin A1 (**5**), hyperin (**6**), isoquercetin (**7**), quercetin (**8**), lupeol (**9**), beta-amyrin (**10**), and alpha-amyrin (**11**). Both procyanidin B3 and A1 exhibited anti-proliferative activity against HepG2 and gastric adenocarcinoma (AGS) cells at IC_50_ values between 38.4 and 41.1 μM and 79.4 and 83.8 μM, respectively. In addition, isoquercetin displayed the strongest anti-proliferative activity against the HepG2, lung carcinoma (A549), and AGS cell at 18.7, 24.6 and 68.5 μM, respectively. Among the triterpenoids, only lupeol showed the inhibitory activity against all tested tumor cell lines at IC_50_ values between 72.9 and 146.8 μM. Furthermore, procyanidins B3, A1 and isoquercetin displayed moderate anti-bacterial activity against *Staphylococcus aureus*. In conclusion, this study provides background information on the exploitation of *V. emarginatum* as a potential natural anti-cancer and anti-bacterial agent in pharmaceutical research.

## 1. Introduction

The genus *Vaccinium* comprising approximately 450 species in the family *Ericaceae*, is a common and widespread genus of morphologically diverse terrestrial shrubs. *Vaccinium* is a class of berry fruits including blueberries (*V. corymbosum*), bilberries (*V. myrtillus*), lingonberries (*V. vitis-idaea*), and cranberries (*V. macrocarpon*), which are consumed by humans [1]. It has been reported as one of the rich sources of naturally occurring bioactive phytochemicals, including phenolic acids, flavonoids, anthocyanins, proanthocyanidins and tannins [2,3,4,5]. Vaccinium berries are well-known for their high antioxidant metabolite contents, which have high potential in anti-oxidant [6,7], anti-bacterial [7], anti-inflammatory [8,9], anti-diabetic [10,11], and anti-proliferative activities [12,13]. According to their broad-spectrum activities, various Vaccinium leaf and berry extracts have been developed as commercial products for dietary supplements [14,15]. 

**Figure 1:**
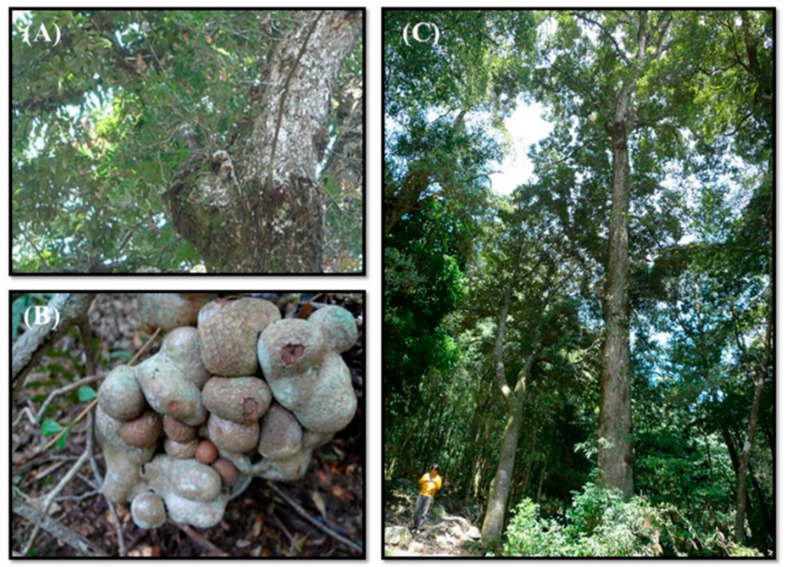
*V. emarginatum* Hayata is an epiphytic shrub grow on the branch of the tree (**A**) in the broad-leaved forests (**C**) with enlarged root nodules (**B**) storing the water and nutrients.

*Vaccinium emarginatum* Hayata (*Ericaceae*) is an endemic species distributed across central mountain regions in Taiwan at an elevation of 1500–3000 m [16]. Unlike other *Vaccinium* species, it is an epiphytic shrub growing on the branches of trees in the broad-leaved forests with enlarged root nodules storing the water and nutrients (Figure 1). In Taiwan, the root nodules are used in traditional medicine, which is supposed to be as effective as Lu Rong (the developing antlers of deer; velvet antler). Its leaves are used to treat urinary tract infections and rheumatic pain, while the fruits are used to treat dysentery and enteritis, respectively [9]. 

To date, few phytochemical studies were reported for *V. emarginatum* [5,9]. Previously, one epicatechin derivative and three new flavonoids, emarginin A, B, and C, were isolated from the methanolic extract of the whole plants of *V. emarginatum*. Emarginin C exhibited moderate anti-inflammatory activity [5]. Recently, the cytotoxic and anti-inflammatory activity of terpenoids from the whole plant of *V. emarginatum* was also investigated [9]. Although those studies indicated the high potential of *V. emarginatum* in anti-inflammatory and cytotoxicity against prostate cancer cells, the anti-infective activity on human pathogens and anti-proliferative effect on tumor cells are still unknown. The object of this study is to evaluate the anti-proliferative and anti-bacterial activities of different chemical fractions from *V. emarginatum* methanol extracts and to characterize its phytochemical profile. The chemical composition of bioacitve fraction was further analyzed by LC-ESI-MS/MS, and the chemical constitutes were isolated and structurally identified by NMR. Finally, the anti-proliferative activities of isolated compounds were determined by measuring the IC_50_ of hepatocellular carcinoma (HepG2), lung carcinoma (A549) and gastric adenocarcinoma (AGS). The anti-bacterial activities of isolated compounds were also tested against seven bacterial pathogens by minimum inhibitory concentration (MIC) methods. It is suggested that all of these isolated compounds may have the potential in clinical treatment as therapeutic agents in the future.

## 2. Results

### 2.1. Biological Activity of V. emarginatum Extract

#### 2.1.1. Anti-Proliferation Activity of the *V. emarginatum* Extract on HepG2 Cell Line

**Figure 2:**
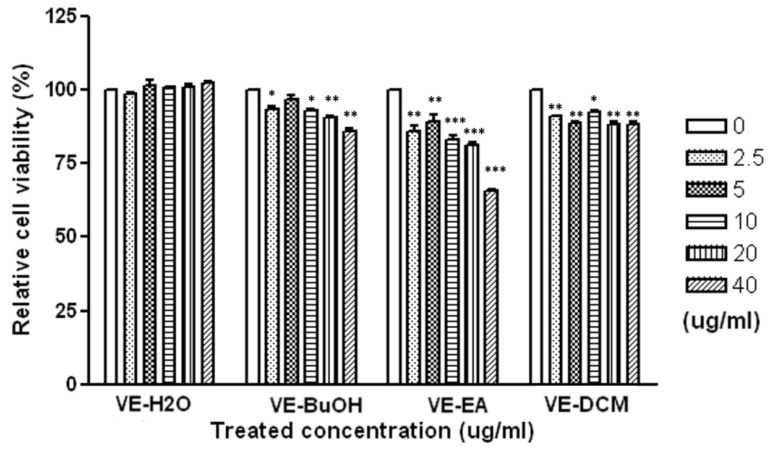
The anti-proliferative activity of different fractions from *V. emarginatum* on human hepatocellular carcinoma cells (HepG2 cell line). The HepG2 cells were treated with a different concentration from 0 to 40 ug/mL of DCM (dichloromethane), EA (ethyl acetate), BuOH (n-butanol), and water fractions of *V. emarginatum*. * *p* < 0.05; ** *p* < 0.01; *** *p* < 0.001.

The cytotoxic effect of different fractions of *V. emarginatum* extract was examined on the HepG2 cell line over 72 h. Aliquots of DCM (dichloromethane), EA (ethyl acetate), BuOH (n-butanol), and water fractions (for a concentration range from 2.5 to 40 μg/mL) were evaluated for the anti-proliferative activity. The DCM and BuOH fractions displayed moderate cytotoxicity, with a relative cell viability of 91.6% to 93.9%, and 86.3% to 96.9% on HepG2 cells, respectively, in comparison with the control (Figure 2). It was revealed that the EA fractions exhibited dose-dependent inhibitory activities on the HepG2 cell, with a relative cell viability of 65.9% to 85.6%. The results show that the HepG2 cell was potentially inhibited by the EA fraction of *V. emarginatum* extract.

#### 2.1.2. Anti-Bacterial Activity of the *V. emarginatum* Extract by the Disc Diffusion Method

Seven strains of pathogens, including Staphylococcus aureus (ATCC 43300), Enterococcus faecalis (ATCC 29212), Listeria monocytogenes (ATCC 7644), Bacillus cereus (ATCC 9139), Escherichia coli (ATCC 35150), Salmonella enterica (ATCC 13311), and Pseudomonas aeruginosa (ATCC 27853), were selected to evaluate the anti-bacterial activity. Among all of the partitioned fractions, ethyl acetate (EA) fractions and the butanol (BuOH) displayed higher anti-bacterial activity against all tested pathogens. The BuOH fraction presented a slightly lower anti-bacterial activity than the EA fraction. As shown in Table 1, the EA fraction of V. emarginatum extract showed anti-bacterial activity against the test strains of S. aureus, E. faecalis, L. monocytogens and B. cereus with average inhibition zones of 14, 7, 9 and 10 mm by using the disc diffusion assay. The EA-15 fraction revealed the most significant and broad anti-bacterial spectrum against the strains of S. aureus, E. faecalis, L. monocytogens and B. cereus, with average inhibition zones of 18, 9, 12 and 14 mm, respectively. 

### 2.2. LC-ESI-MS/MS Analysis of EA Fraction of V. emarginatum Extract

**Figure 3:**
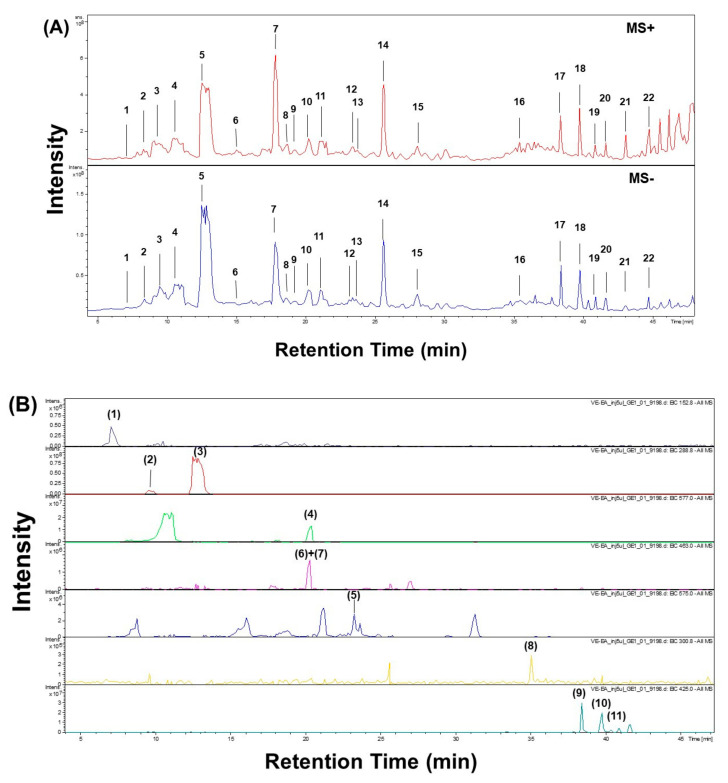
(**A**). LS-ESI-MS chromatograms of EA fraction of *V. emarginatum* extract in positive (+) and negative (−) ionization modes. (**B**). Extraction Ion Chromatograms of Isolated Compounds (**1–11**) in negative (−) ionization modes.

The chromatographic profile EA fraction of *V. emarginatum* extract was determined through LC-ESI-MS/MS analysis (Figure 3). The data of both positive and negative MS and MS^2^ spectra were obtained and the component of the EA fraction was identified partially according to the literature. 

As shown in Table 2, twenty-two peaks can be characterized and twenty peaks can be identified by comparing the spectrum data to references reported previously. For example, loss of CO_2_ was observed for dihydroxyisophthalic acid and protocatechuic acids, giving the [M−H—44]^—^ as a characteristic ion [17]. As described by Sanchez-Rabaneda F. et al., catechin and epicatechin generated a loss of a −CH_2_CHOH− group (*m/z* 245) [17]. Peak 11 showed free (epi)catechin ions at *m/z* 289, indicating condensed tannin dimers consisting of (Epi)afzelechin-(Epi)catechin at *m/z* 561 [18].

For Peaks 2, 4 and 10, the [M−H]^−^ ion at *m/z* 577 suggested the procyanidin dimer with a B-type linkage. Elimination of a phloroglucinol molecular from the B-type dimer resulted from the ion products of MS^2^ at m/z 451 [M−H—126]^−^. Ions at *m/z* 407 [M−H—170]^−^ and *m/z* 289 [M−H—288]^−^ were all suggested by the B-type procyanidin dimmer (Table 2) [19]. Those peaks were identified as procyanidin B1, B2 and B3 according to the literature and isolated chemicals [19]. For Peak 6 and 13 in Figure 3, the [M−H]^−^ ion at *m/z* 863 (M.W. 864) suggested an A-type procyanidin trimer with an interflavanoid linkage (Table 2). According to the ion products of the MS^2^ of Peak 6, ions at the *m/z* 711 [M−H—152]^−^, *m/z* 693 [M−H—152 − 18]^−^ compound in Peak 6 were identified as A-type procyanidin trimer [19]. Furthermore, Peak 13 was identified as procyanidin A1 according to the literature and isolated chemicals. 

Fragmentation pattern of Peaks 9, 11, 15 and 16 displayed the diagnostic ions at m/z 301, suggesting that these compounds were quercetin and quercetin derivatives [20]. For the hyperin and isoquercitin, the spectra showed the ion related to the deprotonated aglycone [A − H] ^−^, which is formed by loss of the galactose or glucose moiety from the flavonol glycosides [17]. The fragmentation pattern of Peaks 17, 18, 19 and 20 contained a negative ion at *m/z* 425 [M−H]^−^ and a positive ion at 427 *m/z* 427 [M + H]^+^, suggesting the structure of C_30_H_50_O. Observed ions’ *m/z* corresponds to [M−H—H_2_O]^−^, [M−H—2H_2_O]^−^, and [M−H—H_2_O − CH_2_CHOH] ^−^ at 407, 389 and 363, indicating the structure of triterpenoid. In addition, the peaks were identified as lupeol, β-amyrin, α-amyrin and friedelin according to the retention time, the literature and the isolated chemicals in this study [21,22,23].

### 2.3. Isolation and Identification of Chemical Components from EA Fraction of V. emarginatum Extraction

**Figure 4:**
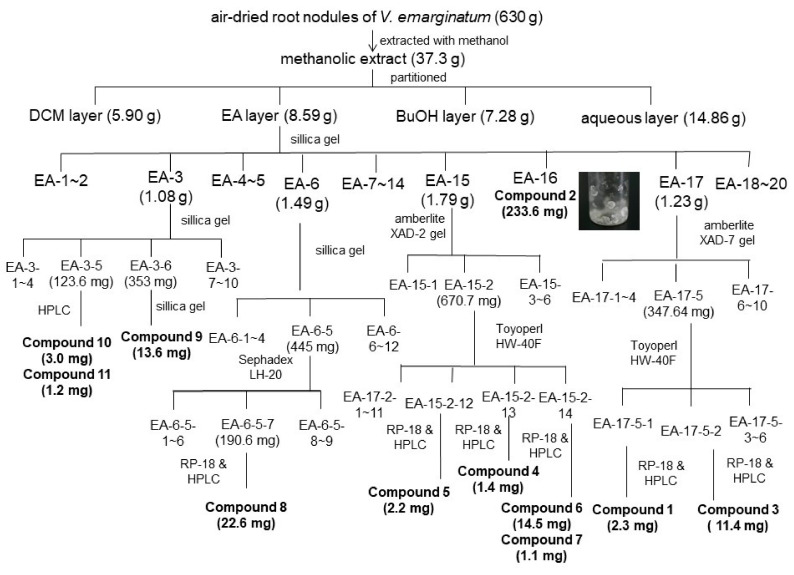
Purification flow chart of chemical constituents isolated from root nodules of *V. emarginatum.*

**Figure 5:**
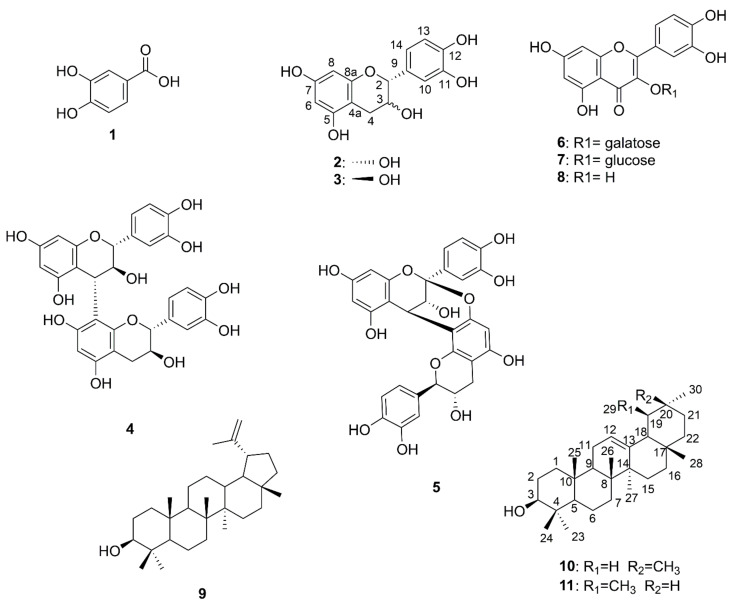
Chemical structures of isolated compounds **1**–**11**.

The anti-proliferative and anti-bacterial constitutes of the most effective fractions are in the EA portion. The EA-soluble fraction (4.59 g) was applied to silica gel column chromatography using a gradient solvent system (n-hexane/EA/MeOH). As shown in Figure 4, collected fractions were checked by TLC and combined into 20 main subfractions (EA-1~EA-20). Fraction EA-3, EA-6, EA-17, EA-18 and EA-19 were isolated by using column chromatography and HPLC to obtain 11 pure compounds: Compound **1** (2.3 mg), **2** (233.6 mg), **3** (11.4 mg), **4** (1.4 mg), **5** (2.2 mg), **6** (14.5 mg), **7** (1.1 mg), **8** (22.6 mg), **9** (13.6 mg), **10** (3.0 mg) and **11** (1.2 mg). Purified compounds were applied to spectroscopic identification by using ^1^H-NMR, ^13^C-NMR and mass analysis. All of the carbon and proton signals were assigned based on the correlation of heteronuclear multiple bond correlation (HMBC) and heteronuclear multiple-quantum (HMQC). The chemical structures of isolated phenolic (**1**), flavonoids (**2**–**3**, **8**), procyanidins (**4–5**), flavonoid glycosides (**6–7**), and triterpenoids (**9**–**11**) were illustrated in Figure 5.

The NMR spectrums and mass data of compounds (**1–11**) from the root nodules of *V. emarginatum* were compared with previous reports. Eleven chemicals were identified as protocatuchuic acid (**1**) [25], epicatechin (**2**) [24,25], catechin (**3**) [24,25], procyanidin B3 (**4**) [24,26,27], procyanidin A1 (**5**) [24,26,27], hyperin (**6**) [28], isoquercetin (**7**) [29], quercetin (**8**) [24,30], lupeol (**9**) [30,31], β-amyrin (**10**) [31,32], and α-amyrin (**11**) [31], respectively (Figure 5). 

### 2.4. Anti-Proliferative Activity of Isolated Compounds

Anti-proliferative activities of Compounds **1–11** were determined by measuring the IC_50_ of hepatocellular carcinoma (HepG2), lung carcinoma (A549) and gastric adenocarcinoma (AGS). As shown in Table 3, none of the phenolic acid and flavonoids have the anti-proliferative activity. Both of the procyanidins (Compound **4** and **5**) exhibited anti-proliferative activity against HepG2 at IC_50_ values between 38.4 and 41.1 μM. The AGS cells were moderately inhibited by procyanidins at IC_50_ values between 79.4 and 83.8 μM. In addition, Compound **7** displayed the strongest anti-proliferative activity against the HepG2, A549, and AGS cell proliferation at 18.7, 24.6 and 68.5 μM, respectively. Among the triterpenoids (Compound **9–11**), only Compound **9** showed the inhibitory activity against all the tested tumor cell lines at IC_50_ values between 72.9 and 146.8 μM. 

### 2.5. Anti-Bacterial Activity of Isolated Compounds

The minimum inhibitory concentration (MIC) method was applied for an anti-bacterial activity exploration of isolated compounds. As shown in Table 4, only procyanidins dimer (Compound **4** and **5**) and one flavonoid glycoside (Compound **7**) displayed moderate anti-bacterial activity against *S. aureus*. Most of the triterpenoids displayed non-anti-bacterial activities against all tested pathogens, expect for Compound **7**, which showed moderate activity against *E. faecalis* at a concentration of 64 μg/mL. In addition, only Compound **5** showed moderate antimicrobial activities against *B. cereus* and *L. monocytogenes*. None of the isolated compounds had anti-bacterial activities against *S. enterica* in this study.

## 3. Discussion

In this study, the biological activities of different chemical fractions from *V. emarginatum* methanol extracts were evaluated. The results indicate that the EA fraction showed pronounced anti-cancer and anti-bacterial activity, and its phytochemical profile was further analyzed by LC-ESI-MS/MS. Twenty-two peaks were identified as five flavonoids, three flavonol glycosides, six procyanidins, one phenolic acid, five triterpenoids and two unknown chemicals. LC-ESI-MS/MS with negative ion detection had been dramatically used for tentative identification of a variety of phenolic compounds in fruits [3,17,19], flowers [20], and traditional herb medicine [18,22]. LC-ESI-MS/MS is a great analytical tool because of its high selectivity for analyzing complex components in plants. Moreover, negative ESI-MS provided a qualitative analysis useful in the screening of phenolics, flavonoids, and procyanidins. It provides simple MS/MS spectra in which it was possible to observe the loss of units more easily in contrast to positive ESI-MS fragmentation with Na^+^ adducts [18,33]. 

Triterpenoids have been analyzed for a long period and are still widely detected and identified using gas chromatography mass spectrometry (GC-MS) [34,35]. Most triterpenoids are lacking a chromophore group, for example, neutral triterpenes have only one hydroxyl group on their structure, such as lupeol and amyrin, which are not easily ionized by ESI. Although liquid chromatography is more suitable for non-volatile chemicals, the triterpenoids founded in this study can be identified by both negative and positive LC-ESI-MS/MS and structurally identified by NMR. 

Previously, the new flavonoid emarginin C, which was isolated from the whole plants of *V. emarginatum*, exhibited anti-inflammatory activity with an IC_50_ value of 27.99 μM [5]. In addition, the triterpenoid coumaroyl and feruloyl esters exhibited strong cytotoxicity against PC-3 prostate cancer cells, with 85.6–90.2% inhibition at 10.0 μg/mL [9]. In this study, the anti-proliferative activities of isolated compounds were determined by measuring the IC_50_ of hepatocellular carcinoma (HepG2), lung carcinoma (A549) and gastric adenocarcinoma (AGS). The results indicate that isolated procyanidin B3 and A1 are the promising lead compounds of *V. emarginatum* for anti-proliferative activities. Other procyanidins, such as procyanidin A2, showed an IC_50_ value of 62.19 μg/mL on human hepatoma HepG2 cells [36]. All of these findings suggest that procyanidins may act as anti-proliferative agents in human hepatoma cells.

*Vaccinium* species are perennial and low-growing herbs found in the temperate climate regions. The berries of *Vaccinium* contain abundant flavonoids, including flavonol glycosides, anthocyanins, flavan3-ols, and procyanidins [37]. Among flavonoids, procyanins are the greatest contributors to the antioxidant capacity in the genus *Vaccinium* [38]. Most of the wild and cultivated cranberries contain both A- and B-type procyanidins. In this study, the EA fraction of *V. emarginatum* methanol extract also contained both dimeric and trimeric procyanidins. Peak 2, 4 and 10 were identified as B-type procyanidins B1, B2 an B3 according to the data of retention time, mass spectrum and MS/MS ion [19,24]. In addition, A- type procyanidins, including dimer A1 and three unknown trimers, were also found in this study. The procyanidin trimer with more OH groups in one benzene unit may increase the antioxidant activity significantly [26]. Flavonoids with a dihydroxy structure on the B ring have been shown to be effective in biological function [39]. In the group of flavonoids, this catechol unit is found in catechins, epicatechin, quercetin, and procyanidins. All of these flavonoids were also found in the root nodule of *V. emarginatum*, indicating that not only the berries but also the root nodule of *V. emarginatum* can be a great source of procyanidins.

In this study, the anti-bacterial activities of isolated compounds were also tested against seven bacterial pathogens by minimum inhibitory concentration (MIC) methods. The determination of MIC against Gram-positive bacteria gave a value of 64 μg/mL for procyanidin B3 (**4**) and A1 (**5**). Previous reports revealed a moderate for certain procyanidins against *Bacillus cereus*, *Klebsiella pneumoniae*, *Proteus vulgaris* and *Streptococcus pyogenes* at concentrations below 100 μg/mL [40]. In addition, procyanidin B2, which was also found in the EA fraction by LC-ESI-MS/MS analysis in this study, generated anti-bacterial activity against *S. aureus* at an MIC value of 100 μg/mL [41]. All of these results indicate that procyanidin dimers displayed moderate antimicrobial activity against certain pathogens. As a potential anti-cancer agent, procyanidin has been shown to inhibit the proliferation of various cancer cells [26,42]. Moreover, several assays demonstrate potential biological functions of procyanidins, including antioxidant, radical-scavenging properties [26], and enzyme inhibiting [43] has also been reported. It is suggested that procyanidin might be the valuable group of *V. emarginatum* as a source of promising compounds for the development of future pharmacological applications. 

## 4. Materials and Methods

### 4.1. General Information

Column chromatography (CC) was carried out by using Sephadex LH-20 (Amersham Pharmacia Biotech, Uppsala, Sweden), LiChroprep RP-18 (Merck, Darmstadt, Germany), XAD-2 (Sigma-Aldrich, St. Louis, MO, USA), XAD-7 (Sigma-Aldrich, St. Louis, MO, USA), Toyopearl HW-40F (Tosoh Bioscience, Tokyo, Japan), and silica gel 60 (Merck, 230–400 mesh, Darmstadt, Germany). Silica gel plates (Merck, F254, 0.25 mm) and RP-18 plates (Merck, F254s, 0.25 mm) were used for thin-layer chromatography (TLC) analyses. Purification was processing with a semi-preparative reversed-phase column (Merck, Hibar Purospher RP-18e, 5 μm, 250 × 10 mm) on the Hitachi HPLC (L-2130 pump with a L-2420 UV-vis detector). 

The NMR spectra data were measured on an Avance NEO 400 spectrometer (Bruker Daltonics Inc., Billerica, MA, USA) and an Agilent Technologies DD2 600 spectrometer (Agilent, Santa Clara, CA, USA). ESI-MS spectra were performed on a Bruker Daltonics Esquire HCT spectrometer (Bruker Daltonics Inc., Billerica, MA, USA).

### 4.2. Plant Materials

The root nodules of *Vaccinium emarginatum* Hayata were collected from cloud forests in Jan 2010 and Feb 2010, at the Dasyueshan site (24°14′13.43′′ N, 120°57′10.86′′ E at 1939 m asl.; annual rainfall: 2000–3000 mm) in Taiwan. *V. emarginatum* was identified by the Key Laboratory of the High Altitude Experimental Station in the Taiwan Endemic Species Research Institute. The voucher specimen (2010-0120-Wang) was preserved in the Lab of Chemical Ecology, Research Center for Biodiversity, China Medical University. 

### 4.3. Liquid Chromatography-Electrospray Ionization/Tandem Mass Spectrometry (LC-ESI-MS/MS)

LC mass analysis for the chemical components in the EA fraction was applied to LC-ESI-MS/MS) analysis (Esquire HCT, Bruker Daltonics, Billerica MA, USA). At a flow rate of 0.25 mL/min, the Atlantis T3 RP-18 column (150 × 2.1 mm; 3 μm; Waters, Milford, MA, USA) was eluted with Buffer A (distilled water/acetonitrile/formic acid; 95/5/0.1, *v/v/v*) and Buffer B (acetonitrile/formic acid; 100/0.1, *v/v*) at 25 °C. Elution started initially with 100% Buffer A, followed by a linear increase in Buffer B to 10% from 0 to 5 min, and maintained in 10% Buffer B for another 5 min. A secondary linear increase in Buffer B to 20% was carried out from 10 to 20 min and maintained in 20% Buffer B for another 10 min. From 30 to 35 min, the column was further eluted with a third linear increase in Buffer B to 60% and maintained for another 5 min. A final linear increasing of Buffer B was carried out from 60% to 95% and maintained for 5 min from 40 to 45 min. The column was finally equilibrated with Buffer A for another 10 min. The ion source temperature is maintained at 100 °C and the dry temperature is 365 °C. The flow rate of N_2_ desolvation gas is sustained at 12 L/min. Product ion scans for mass were accomplished by low-energy collision (20 eV) using argon as the collision gas. MS spectrum data of both positive and negative ionization mode were collected. All data were analyzed by Bruker Daltonics Data software (version 4.0).

### 4.4. Isolation and Identification of Chemical Constitutes from V. emarginatum Extract

Six kilograms of *V. emarginatum* root nodules was air-dried (630 g) and extracted with methanol thrice following the standard extraction procedures. The methanolic extract was concentrated to gain 37.3 g dry residue and then partitioned by dichloromethane, ethyl acetate, butanol with H_2_O to the obtained portion of DCM (5.90 g), EA (8.59 g), BuOH (7.28 g), and an aqueous layer (14.86 g), sequentially.

Bioassay-guided chromatography was carried out for anti-proliferative and anti-bacterial compound isolation (Figure 2; Table 1). The EA portion was subjected to a silica gel column in gradient elution of mixture solvent composed of hexane-ethyl acetate and led to 20 fractions. Fraction EA-3 (1.08 g) was separated via silica gel column with a hexane-ethyl acetate mixture as the eluent to obtain 10 subfractions (EA-3-1~EA-3-10) and Compound **9** (13.6 mg), **10** (3.0 mg) and **11** (1.2 mg) were purified from subfraction EA-3-5 and EA-3-6. Fraction EA-6 was further fractionated by another silica gel column with a hexane-ethyl acetate mixture (5:1) to give 12 subfractions. Compound 8 was isolated from fraction 6-5-7 by reverse-phase C-18 chromatography in gradient elution of MeOH–H_2_O (60% to 100%). Compounds **4** (1.4 mg), **5** (2.2 mg), **6** (14.5 mg) and **7** (1.1 mg) were isolated form the effective fractions 15-2-12, 15-2-13 and 15-2-14 by reverse-phase C-18 chromatography in gradient elution of MeOH–H2O (40% to 100%). Compound **2** was crystallized from subfraction EA-16 fractionated by silica gel column with a hexane-ethyl acetate mixture (1:8). In addition, Compound **1** (2.3 mg) and **3** (11.4 mg) were isolated from the fraction of EA-17 (1.23 g), by XAD-7, HW-40F and RP-18 chromatography in gradient elution of MeOH–H2O (10% to 100%). Purified compounds were subjected to spectroscopic identification using ^1^H-NMR and ^13^C-NMR (Agilent Technologies DD2 600) and Mass (Bruker Daltonics Esquire HCT). All isolated compounds were identified by comparison of spectra data with literature reported previously. Detail structure elucidation of isolated compounds in this study was described in Supplementary Materials.

Protocatechuic acid (**1**). White amorphous powder; ESI-MS: [M−H]^–^, 152.7 *m*/*z*, (calcd for C_7_H_5_O_4_: 153.1). ^1^H-NMR (400 MHz, CD_3_OD): δH 7.42 (1H, d, *J* = 1.8 Hz, H-2), 7.40 (1H, dd, *J* = 7.8, 2.0 Hz, H-5), 6.79 (1H, d, *J* = 8.0 Hz, H-6). 

Epicatechin (**2**): White amorphous powder; ESI-MS: [M−H]^–^, 288.8 *m*/*z*, (calcd for C_15_H_13_O_6_: 289.2); ^1^HNMR (400 MHz, CD_3_OD): δH 6.96 (1H, d, *J* = 1.6 Hz, H-10), 6.76 (1H, d, *J* = 8.8 Hz, H-14), 6.74 (1H, dd, *J* = 2.0, 8.8 Hz, H-13), 5.93 (1H, d, *J* = 2.0 Hz, H-8), 5.90 (1H, d, *J* = 2.4 Hz, H-6), 4.80 (1H, s, H-2), 4.15 (1H, d, *J* = 3.2, H-3), 2.86 (1H, dd, *J* = 8.8, 16.8 Hz, H-4 ax), 2.73 (1H, dd, *J* = 2.8, 16.8 Hz, H-4 eq); ^13^C-NMR (150 MHz, CD_3_OD): δC 157.9 (C-7), 157.6 (C-5), 157.3 (C-8a), 145.8 (C-11), 145.7 (C-12), 132.2 (C-9), 119.3 (C-13), 115.8 (C-14), 115.2 (C10), 100.0 (C-4a), 96.3 (C-6), 95.8 (C-8), 79.8 (C-2), 67.4 (C-3), 29.2 (C-4).

Catechin (**3**): Yellow powder, ESI-MS: [M−H]^–^, 288.8 *m*/*z*, (calcd for C_15_H_13_O_6_: 289.2); ^1^HNMR (400 MHz, CD_3_OD): δH 6.83 (1H, d, *J* = 1.6 Hz, H-10), 6.76 (1H, d, *J* = 8.1 Hz, H-14), 6.70 (1H, dd, *J* = 1.8, 8.1 Hz, H-13), 5.84 (1H, d, *J* = 2.2 Hz, H-8), 5.91 (1H, d, *J* = 2.2 Hz, H-6), 4.55 (1H, dd, *J* = 7.5, H-2), 3.96 (1H, m, H-3), 2.83 (1H, dd, *J* = 5.4, 16.1 Hz, H-4), 2.49 (1H, dd, *J* = 8.1, 16.1 Hz, H-4); ^13^C-NMR (150 MHz, CD_3_OD): δC 157.7 (C-7), 157.5 (C-5), 156.8 (C-8a), 146.2 (C-11), 146.2 (C-12), 132.1 (C-9), 120.0 (C-13), 116.0 (C-14), 115.2 (C10), 100.8 (C-4a), 96.3 (C-6), 95.5 (C-8), 82.8 (C-2), 68.8 (C-3), 28.5 (C-4).

Procyanidin B3 (**4**): White amorphous powder; ESI-MS: [M−H]^–^, 577.1 *m*/*z*, (calcd for C_30_H_25_O_12_: 577.5); ^1^H-NMR (CD_3_OD, 400 MHz): δH 6.94 (1H, d, *J* = 1.8 Hz, H-10), 6.76 (1H, dd, *J* = 6.0, 1.8 Hz, H-14), 6.67 (1H, d, *J* = 7.8 Hz, H-13), 5.87 (1H, d, *J* = 2.4 Hz, H-6), 5.78 (1H, d, *J* = 2.4 Hz, H-8), 4.36 (1H, d, *J* = 5.4 Hz, H-3), 4.39 (1H, d, *J* = 7.8 Hz, H-4), 4.24 (1H, d, *J* = 7.8 Hz, H-2), 4.53 (1H, d, *J* = 7.2 Hz, H-2′), 3.77 (1H, m H-3′), 2.48 (1H, dd, *J* = 16.2, 7.8 Hz, H-4′α), 2.76 (1H, dd, *J* = 16.2, 5.4 Hz, H-4′β), 6.57 (1H, d, *J* = 1.8 Hz, H-10′), 6.82 (1H, dd, *J* = 6.0, 2.4 Hz, H-14′), 6.65 (1H, d, *J* = 8.4 Hz, H-13′), 6.06 (1H, s, H-6′); ^13^C-NMR (CD_3_OD, 150 MHz): δC 84.0 (C-2), 73.6 (C-3), 38.5 (C-4), 107.1 (C-4a), 157.2 (C-5), 97.5 (C-6), 157.4 (C-7), 96.2 (C-8), 158.6 (C-8a), 132.4 (C-9), 116.1 (C-10), 146.1 (C-11), 146.3 (C-12), 116.0 (C-13), 121.0 (C-14), 82.9 (C-2′), 68.5 (C-3′), 28.4 (C-4′), 100.4 (C-4′a), 154.9 (C-5′), 95.5 (C-6′), 155.7 (C-7′), 108.3 (C-8′), 155.6 (C-8′a), 132.1 (C-9′), 115.1 (C-10′), 146.1 (C-11′), 146.3 (C-12′), 115.9 (C-13′), 120.1 (C-14′).

Procyanidin A1 (**5**): White amorphous powder; ESI-MS *m*/*z* 575.1 [M−H]^–^ (Calcd for C_30_H_23_O_12_: 575.5). ^1^H-NMR (CD_3_OD, 600 MHz) δH 4.06 (1H, d, *J* = 4.2 Hz, H-3), 4.23 (1H, d, *J* = 3.6 Hz, H-4), 5.95 (1H, d, *J* = 2.4 Hz, H-6), 6.06 (1H, d, *J* = 2.4 Hz, H-8), 7.12 (1H, d, *J* = 1.8 Hz H-10), 6.81 (1H, d, *J* = 8.4 Hz, H-13), 7.01 (1H, dd, *J* = 8.4, 2.4 Hz, H-14), 4.72 (1H, d, *J* = 7.8 Hz H-2′), 4.14 (1H, m, H-3′), 2.57 (1H, dd, *J* = 16.2, 8.4 Hz, H-4′α), 2.94 (1H, dd, *J* = 16.2, 5.4 Hz, H-4′β), 6.08 (1H, s, H-6′), 6.91 (1H, s, H-10′), 6.81 (1H, s, H-13′), 6.81 (1H, d, *J* = 8.4 Hz, H-14′); ^13^C-NMR (CD_3_OD, 150 MHz) δC 100.3 (C-2), 67.8 (C-3), 29.2 (C-4), 104.0 (C-4a), 156.8 (C-5), 98.1 (C-6), 158.1 (C-7), 96.5 (C-8), 154.2 (8a), 132.3 (C-9), 115.6 (C-10), 146.8 (C-11), 145.6 (C-12), 116.3 (C-13), 119.8 (C-14), 84.5 (C-2′), 68.1 (C-3′), 29.0 (C-4′), 103.1 (C-4′a), 156.1 (C-5′), 96.5 (C-6′), 152.2 (C-7′), 106.8 (C-8′), 151.4 (C-8′a), 130.5 (C-9′), 115.7 (C-10′), 146.8 (C-11′), 146.3 (C-12′), 115.7 (C-13′), 120.7 (C-14′).

Hyperin (**6**): Amorphous yellow powder; ESI-MS *m/z* 462.9 [M−H]^–^ (Calcd for C_21_H_19_O_12_: 463.3). ^1^H-NMR (DMSO-d6, 400 MHz): 7.66 (2H, dd, *J* = 2.2, 8.4 Hz, H-6′), 7.53 (1H, d, *J* = 2.4 Hz, H-2′), 6.82 (1H, d, *J* = 8.4 Hz, H-5′), 6.40 (1H, d, *J* = 2.0 Hz, H-8), 6.20 (1H, d, *J* = 2.0 Hz, H- 6), 5.37 (1H, d, *J* = 7.6 Hz, gatactosyl H-I”); ^13^C-NMR (DMSO-d6, 100 MHz): δC 177.7 (C4), 164.4 (C-7), 161.4 (C-5), 156.5 (C-2, C-9), 148.7 (C-4′), 145.0 (C-3′), 133.7 (C-3), 122.2 (C-6′), 121.3 (C-1′), 116.2 (C-5′), 115.4 (C-2′), 104.1 (C-10), 102.1 (C-1”), 98.9 (C-6), 93.7 (C-8), 76.1 (C-5”), 73.4 (C-3”), 71.4 (C-2”), 68.2 (C-4”), 60.4 (C-6”).

Isoquercetin (Quercetin-3-O-D-glucopyranoside) (**7**): Amorphous yellow powder, ESI-MS *m/z* 463.2 [M−H]^–^ (Calcd for C_21_H_19_O_12_: 463.3). ^1^H-NMR (DMSO-d6, 400 MHz): δH 7.58 (2H, d, *J* = 8.8 Hz, H-2′ and H-6′), 6.85 (2H, d, *J* = 8.8 Hz, H-5′), 6.36 (1H, d, *J* = 2.0 Hz, H-8), 6.17 (1H, d, *J* = 2.0 Hz, H-6), 5.25 (1H, d, *J* = 7.2 Hz, glucosyl H-1′′). ^13^C-NMR (DMSO-d6, 100 MHz): δC 177.6 (C-4), 164.5 (C-7), 161.3 (C-5), 156.5 (C-2), 156.3 (C-9), 148.6 (C-4′), 144.9 (C-3′), 133.5 (C-3), 121.7 (C-6′), 121.2 (C-1′), 116.3 (C-5′), 115.3 (C-2′), 104.1 (C-10), 101.0 (C-1′′), 98.9 (C-6), 93.7 (C-8), 77.6(C-5′′), 76.6 (C-3′′), 74.2 (C-2′′), 70.0 (C-4′′), 61.1 (C-6′′).

Quercetin (**8**): Amorphous yellow powder, ESI-MS *m/z* 301.1 [M−H]^–^ (Calcd for C_15_H_9_O_7_: 301.2). ^1^H-NMR (DMSO-d6, 400 MHz): δH 7.72 (1H, d, *J* = 2.0 Hz, H-2′), 7.62 (1H, dd, *J* = 2.0, 8.4 Hz, H-6′), 6.87 (1H, d, *J* = 8.4 Hz, H-5′), 6.37 (1H, d, *J* = 1.6 Hz, H-8), 6.17 (1H, d, *J* = 2.0 Hz, H-6); ^13^C-NMR (DMSO-d6, 100 MHz): δC 176.4 (C4), 164.4 (C-7), 161.1 (C-5), 157.0 (C-9), 148.2 (C-4′), 147.4 (C-2), 145.5 (C-3′), 136.1 (C-3), 122.5 (C-1′), 120.5 (C-6′), 116.1 (C-5′), 115.4 (C-2′), 103.4 (C-10), 98.6 (C-6), 93.8 (C-8).

Lupeol (**9**): White amorphous powder; ESI-MS *m/z* 425.0 [M−H]^–^ (Calcd for C_30_H_49_O: 425.7); ^1^H-NMR spectrum (600 MHz, CDCl_3_): δH 3.20 (1H, m, H-3), 0.68 (1H, d, *J* = 9.6 Hz, H-5), 2.38 (1H, m, H-19), 4.69 (1H, brs, H-29), 4.57 (1H, brs, H-29), 1.03, 0.97, 0.95, 0.83, 0.79, 0.76 (Me-26, Me-27, Me-23, Me-25, Me-28, Me-24), 1.68 (3H, s, Me-30). ^13^C-NMR spectrum (150 MHz, CDCl_3_): δC: 150.9 (C-20), 109.3 (C-29), 78.9 (C-3), 55.2 (C-5), 50.3 (C-9), 48.2 (C-18), 47.9 (C-19), 42.9 (C-17), 42.7 (C-14), 40.7 (C-8), 39.9 (C-22), 38.8 (C-13), 38.6 (C-4), 38.0 (C-1), 37.1 (C-10), 35.5 (C-16), 34.2 (C-7), 25.0 (C-2), 20.8 (C-11), 27.4 (C-12), 27.9 (C-15), 29.8 (C-21), 29.6 (C-23), 19.2 (C-30), 18.2 (C-6), 17.9 (C-28), 16.1 (C-25), 15.9 (C-26), 15.3 (C-24),14.5 (C-27).

β-Amyrin (**10**): Colorless solid; ESI-MS *m/z* 425.1 [M−H]^–^ (Calcd for C_30_H_49_O: 425.7); ^1^H-NMR spectrum (600 MHz, CDCl_3_): δH 3.23 (1H, dd, *J* = 10.4, 4.8 Hz, H-3), 0.74 (1H, dd, J = 12.0, 1.2 Hz, H-5), 1.56 (1H, dd, *J* = 7.8, 1.8 Hz, H-9), 5.18 (1H, t, *J* = 3.6 Hz, H-12), 1.94 (1H, dd, *J* = 14.4, 4.8 Hz, H-18), 1.13 (3H, s, Me-27), 0.99, 0.96, 0.93, 0.88, 0.87, 0.83, 0.79 (Me-23, Me-26, Me-25, Me-29, Me-30, Me-28, Me-24). ^13^C-NMR spectrum (150 MHz, CDCl_3_): δC: 145.2 (C-13), 121.7 (C-12), 79.0 (C-3), 55.1 (C-5), 47.6 (C-9), 47.2 (C-18), 46.8 (C-19), 41.7 (C-14), 39.7 (C-8), 38.7 (C-4), 38.5 (C-1), 37.1 (C-22), 36.9 (C-10), 34.7 (C-21), 33.3 (C-29), 32.6 (C-7), 32.4 (C-17), 31.1 (C-20), 28.3 (C-28), 28.0 (C-23), 27.2 (C-2), 26.9 (C-16), 26.1 (C-15), 25.9 (C-27), 23.6 (C-30), 23.5 (C-11), 18.3 (C-6), 16.7 (C-26), 15.5 (C-24), 15.5 (C-25).

α-Amyrin (**11**): Colorless solid; ESI-MS *m/z* 425.0 [M−H]^–^ (Calcd for C_30_H_49_O: 425.7); ^1^H-NMR spectrum (600 MHz, CDCl_3_): δH 3.30 (1H, dd, *J* = 11.4, 5.4 Hz, H-3), 0.74 (1H, dd, J = 12.0, 1.2 Hz, H-5), 5.13 (1H, t, *J* = 3.6 Hz, H-12), 1.99 (2H, td, *J* = 13.5, 4.8 Hz, H-15), 1.84 (2H, td, *J* = 13.6, 4.9 Hz, H-16), 1.07, 1.01, 1.00, 0.95, 0.80, 0.79 (Me-27, Me-26, Me-23, Me-24, Me-28, Me-25), 0.78 (3H, d, *J* = 4.8 Hz, Me-29), 0.92 (3H, d, *J* = 6.0 Hz, Me-30). ^13^C-NMR spectrum (150 MHz, CDCl_3_): δC: 139.5 (C-13), 124.4 (C-12), 38.7 (C-1), 28.0 (C-2), 79.0 (C-3), 59.0 (C-18), 55.1 (C-5), 47.7 (C-9), 42.0 (C-14), 41.5 (C-22), 39.9 (C-8), 39.6 (C-19), 39.6 (C-20), 38.7 (C-4), 36.8 (C-10), 33.7 (C-17), 32.9 (C-7), 31.2 (C-21), 28.7 (C-28), 28.1 (C-23), 27.2 (C-15), 26.6 (C-16), 23.3 (C-11), 23.2 (C-27), 21.4 (C-30), 18.3 (C-6), 17.4 (C-29), 16.8 (C-26), 15.6 (C-24), 15.6 (C-25).

### 4.5. Anti-Bacterial Assay

The disc diffusion method was applied to anti-bacterial-guided fractionation. The chemical fractions were dissolved in DMSO at a concentration of 100 μg/μL. According to the standard protocol (Clinical and Laboratory Standards Institute, CLSI) [44], 10 μL of dried extracts was applied to paper discs (6 mm in diameter) and placed on Mueller Hinton agar plates, which were inoculated with overnight-cultured pathogens. The plates were incubated at 37 °C for 18 h. The diameters of the inhibition zones were measured within 24 h, and discs containing DMSO and antibiotics were served as a negative and positive control. At least three independent determinations were repeated. 

The anti-bacterial activity of purified compounds was conducted by the minimum inhibitory concentration (MIC) method. According to the guidelines of the CLSI [45], the MIC values were determined by the broth micro-dilution method. Briefly, all bacterial strains were cultured on nutrient agar and incubated at 37 °C for 24 h. Bacteria were suspended in 0.85% NaCl solution and diluted to give an inoculum with a final density of 5 × 10^5^ cfu/mL. Isolated compounds were dissolved in DMSO at a final concentration of 2560 μg/mL. Two-fold dilutions were made serially in a concentration range from 0.5 to 128 μg/mL. In each 96-well plate, all of the wells were calculated for bacterial growth by spectrophotometer at 590 nm. The lowest concentration that inhibited the growth of the respective pathogen was defined as the MIC value. The antibiotics (ampicillin and tetracycline) were used as a positive control. All tests were carried out in triplicate. 

### 4.6. Anti-Proliferative Assay

Anti-proliferative assay was determined against HepG2 2.2.15 (human hepatocellular carcinoma cells) and A549 cells (human lung adenocarcinoma cell line) using the MTT (3-(4, 5-dimethylthiazol-2yl)-2, 5-diphenyl tetrazolium bromide) assay (Promega, USA). Briefly, HepG2 2.2.15 and A549 cells were treated with 2-fold serial dilutions of the tested compounds ranging from 0.19 to 200 μM for 3 days. After incubation, the medium from the wells was removed carefully. To each well, 50 μL of serium-free medium and 50 μL of MTT solution (5 mg/mL in PBS) were added. The plate was incubated for 3 hours in 37 °C incubator with 5% CO_2_. After incubation, 150 μL MTT solution was added into each well and the plates were shacked on a shaker for 15 min. Mixing well by micropipette may be required to fully dissolve the MTT formazan. The presence of viable cells was visualized by the formation of formazan crystals in purple color. Plates were read at 570 nm for cytotoxicity assay within one hour.

The human gastric adenocarcinoma (AGS cell line) was cultured in RPMI-1640 medium supplemented with antibiotics (100 μg/mL of streptomycin and 100 U/mL of penicillin) and 10% FBS (fetal bovine serum). The AGS cell was treated with compounds ranging from 0.19 to 200 μM for 2 days. The trypan blue exclusion protocol was used for in vitro cytotoxicity assay. In brief, 40 μL of trypan blue was mixed with 10 μL of cell suspension and the numbers of dead cells (stained) and live cells (unstained) were counted using a hemocytometer. After treatment, the cell viability as the percentage of cell survival was recorded and the value of IC_50_ was also calculated. All measurements were performed in triplicate, and epigallocatechin gallate (EGCG), 5-fluorouracil, and doxorubicin were used as the positive control.

## 5. Conclusions

In this study, twenty peaks of EA fraction from *V. emarginatum* extract were identified by LC-ESI-MS/MS analysis, and eleven of them were isolated and identified by NMR analysis. The ability of procyanidin B3, A1, isoquercetrin, quercetin and lupeol to inhibit the tumor proliferative activity means that they have the potential to constitute a valuable group of therapeutic agents in the future.

## Figures and Tables

**Table 1:** The anti-bacterial activity of different fractions from *V. emarginatum* against human pathogens by the disc diffusion method.

	Disc Inhibition Zone (mm)				
Pathogens	DCM *	EA	BuOH	H_2_0	EA-15
*Staphylococcus aureus*	0	14.3 ± 0.6	12.3 ± 0.6	0	18.3 ± 0.6
*Enterococcus faecalis*	0	7.0 ± 0.0	0	0	9.0 ± 0.0
*Listeria monocytogens*	0	9.0 ± 0.0	7.0 ± 0.0	0	12.3 ± 0.6
*Bacillus cereus*	0	10.3 ± 0.6	9 ± 0	0	14.3 ± 0.6
*Escherichia coli*	0	0	0	0	0
*Salmonella enterica*	0	0	0	0	0
*Pseudomonas aeruginosa*	0	0	0	0	0

* DCM: dichloromethane; EA: ethyl acetate; BuOH: n-butanol.

**Table 2:** Characterization of compounds in EA fraction of *V. emarginatum* using LCESI-MS/MS in negative ion mode.

No.	R.T. (min)	Assigned Identity	Mass	M.W.	Negative ESI-MS^n^ *m/z* (% Base Peak)	Reference
1	7.2	Protocatechuic acid	[M−H]^−^: 152.8 [M+H]^+^: 154.9	154	**108.9**(100)	[17]
2	8.3	Procyanidin B1	[M−H]^−^: 577.1 [M+H]^+^: 579.2	578	**407.0**(100), 425.0(63), 288.9(26.5), 451(17.9), 559.1(9.1)	[24]
3	9.5	Catechin	[M−H]^−^: 289 [M+H]^+^: 291	290	270.8(6.3), **244.8**(100), 230.7(8.2), 204.7(38.8), 202.7(22.0), 178.8(10.8), 160.7(8.4)	[17,24]
4	10.8	Procyanidin B2	[M−H]^−^: 577.1 [M+H]^+^: 579.2	578	559.1(8.2), 450.9(15.7), 424.9(74.8), **407.0**(100), 288.9(24.3)	[24]
5	12.4	Epicatechin	[M−H]^−^: 288.8 [M+H]^+^: 290.9	290	270.7(5.3), **244.7**(100), 230.7(11.3), 204.7(47.8), 202.8(31.6), 178.7(13.6), 160.7(7.5)	[17,24]
6	14.7	A-type Procyanidin trimer	[M−H]^−^: 863.1 [M+H]^+^: 865.2	864	**711.1**(100), 411(61.8), 559.1(50.8), 693.1(43.3), 712(23.1), 451(22.9), 694.1(20.9)	[19]
7	17.8	Emarginin B	[M−H]^−^: 358.9 [M+Na]^+^: 383.0	360	**196.7**(100), 152.8(34.3), 134.8(20.3), 108.9(7.8)	[5]
8	18.6	Dihydroxyisophthalic acid	[M−H]^−^: 196.7 [M+H]^+^: 198.9	198	**152.8**(100), 134.8(3.4), 108.9(2.7)	[17]
9	20.1	Hyperin	[M−H]^−^: 462.9 [M+Na]^+^: 487.1	464	**300.7**(100), 178.7(4.7), 150.7(5.7)	[17,20]
10	20.3	Procyanidin B3	[M−H]^−^: 577.1 [M+H]^+^: 579.1	578	559.0(4.8), 450.9(15.9), **425.0**(100), **407.0**(98.4), 288.9(22.8), 286.9(16.1)	[19]
10	20.3	Isoquercetin	[M−H]^−^: 462.9 [M+Na]^+^: 487.1	464	**301**(100), 178.7(4.9), 150.7(4.0)	[17,20]
11	21.3	A-type Procyanidin trimer	[M−H]^−^: 863.3 [M+H]^+^: 865.2	864	**575.0**(100), 711.1(53.4), 699.1(42.9), 693.1(36.7), 576.1(25.4), 713.1(24.4), 821.2(23.9), 803.2(20.1)	[19]
12	23.1	(Epi)afzelechin-(Epi)catechin	[M−H]^−^: 561.1 [M+H]^+^: 563.2	562	**270.8**(100), 451.0(70.4), 423.0(62.8), 435.0(61.2), 408.9(49.3), 298.8(38.7), 288.9(31.8), 324.9(22.7), 282.9(21), 258.8(19.5),	[18]
13	23.3	Procyanidin dimer A1	[M−H]^−^: 575.1 [M+H]^+^: 577.1	576	**423**(100), 449(85.6), 539(42.4), 284.9(35.2), 407(29.1), 423.9(26.1), 288.9(25.1)	[19]
14	25.6	Emarginin C	[M−H]^−^: 373.1 [M+Na]^+^: 397.1	374	**210.7**(100), 373(8.6)	[5]
15	28.0	Quercetin with two glycosides	[M−H]^−^: 591.1 [M+H]^+^: 593.2	592	547.0(17.4), **438.9**(100), 300.9(52.9), 288.9(64.6), 256.8(17.8), 214.7(29.9), 212.7(10.0)	[18,20]
16	35.1	Quercetin	[M−H]^−^: 300.9 [M+H]^+^: 302.9	302	**178.7**(100), 150.7(92.4), 254.9(38.4), 272.8(29), 256.7(10.7)	[17,20]
17	38.4	Lupeol	[M−H]^−^: 425.0 [M+H]^+^: 427.1	426	407(30.8), **363.3**(100), 300.9(17.8), 288(66.6), 256.8(10.3), 244.8(17.5), 214.8(19.1)	[21,22]; this study
18	39.7	β-Amyrin	[M−H]^−^: 425.0 [M+H]^+^: 427.1	426	407(19.3), 389(10.9), 363(80.5), **314.9**(100), 288.8(9.1), 270.9(14.4), 240.7(15.0), 228.8(21.5), 204.7 (11.6), 176.7(10.0)	[21,22]; this study
19	40.3	α-Amyrin	[M−H]^−^: 425.1 [M+H]^+^: 427.1	426	407(50.5), 389(22.3), 381.0(14.2), 362.9(94.3), **314.9**(100), 288.8(14.3), 270.9(14.3), 240.8(18.8), 228.8(28.2), 204.7 (13.0)	[21,22]; this study
20	40.8	Friedelin	[M−H]^−^: 425.1 [M+H]^+^: 427.1	426	407(20.5), **363.3**(100), 300.9(19.9), 288(65.4), 256.8(15), 244.8(14.4), 214.8(21.5)	[21,22,23]
21	42.9	Unknown	[M−H]^−^: 675.4 [M+Na]^+^: 699.4	676	**396.9** (100), 415.0 (50.4), 234.8 (10.5), 397.8 (9.2), 304.8 (5.8), 286.9 (5.6)	
22	44.7	Unknown	[M−H]^−^: 677.4 [M+Na]^+^: 701.4	678	**397.0** (100), 415.0 (34.4), 234.7 (9.4), 304.9 (7.3), 286.8 (6.4)	

**Table 3:** Anti-proliferative activities of isolated compounds on tumor cell lines.

	Anti-Proliferative Activity, IC_50_ (μM)		
Compounds	HepG2 2.2.15	A549	AGS
**1**	>200	>200	>200
**2**	>200	>200	>200
**3**	>200	>200	>200
**4**	41.1 ± 1.3	>200	79.4 ± 2.3
**5**	38.4 ± 1.4	>200	83.8 ± 3.6
**6**	>200	>200	>200
**7**	18.7 ± 0.9	24.6 ± 0.6	68.5 ± 4.5
**8**	64.0 ± 1.4	>200	82.2 ± 4.6
**9**	81.7 ± 1.7	146.8 ± 5.8	72.9 ± 1.7
**10**	>200	174.2 ± 6.1	>200
**11**	>200	>200	>200
EGCG	N.D. *	91.9 ± 1.8	N.D.
5-fluorouracil	62.0 ± 1.1	N.D.	N.D.
Doxorubicin	N.D.	N.D.	11.0 ± 0.6

* N.D.: not determined; **1**: protocatuchuic acid; **2**: epicatechin; **3**: catechin; **4**: procyanidin B3; **5**: procyanidin A1; **6**: hyperin; **7**: isoquercetin; **8**: quercetin; **9**: lupeol; **10**: beta-amyrin; **11**: alpha-amyrin.

**Table 4:** The minimum inhibitory concentration (MIC) value of isolated compounds and antibiotics for different bacterial pathogens.

	MIC (μg/mL)						
	Bacterial Pathogens						
Compounds	*S. aureus*	*E. faecalis*	*L. monocytogenes*	*B. cereus*	*E. coli*	*S. enterica*	*P. aeruginosa*
Ap *	8	2	1	64	4	2	256
Tet	4	2	2	2	1	8	16
**1**	>128	>128	>128	>128	>128	>128	>128
**2**	>128	>128	>128	>128	>128	>128	>128
**3**	>128	>128	>128	>128	>128	>128	>128
**4**	**64**	>128	>128	>128	>128	>128	>128
**5**	**64**	> 128	**64**	**64**	>128	>128	>128
**6**	>128	**128**	>128	**128**	>128	>128	**128**
**7**	**64**	>128	>128	**128**	**128**	>128	**128**
**8**	**128**	>128	**128**	>128	>128	>128	>128
**9**	>128	**64**	>128	>128	>128	>128	>128
**10**	>128	**128**	>128	**128**	>128	>128	>128
**11**	>128	>128	>128	>128	>128	>128	>128

* Ap: ampicillin; Tet: tetracycline; **1**: protocatuchuic acid; **2**: epicatechin; **3**: catechin; **4**: procyanidin B3; **5**: procyanidin A1 c; **6**: hyperin; **7**: isoquercetin; **8**: quercetin; **9**: lupeol; **10**: beta-amyrin; **11**: alpha-amyrin.

## Data Availability

The data presented in this study are available on request from the corresponding author.

## Supplementary Materials

The following are available online at https://www.mdpi.com/article/10.3390/ph14111098/s1, Figures S1–S27 NMR spectral (1D or 2D for isolated compounds) and MS/MS data.
