# Supplementary material for: HPLC/ESI-MS and NMR Analysis of Chemical Constitutes in Bioactive Extract from the Root Nodule of Vaccinium emarginatum
Source: Pharmaceuticals (Basel). 2021 Oct 28;14(11):1098. doi: 10.3390/ph14111098 (PMC8622236; doi:10.3390/ph14111098)
Supplement: Supplementary file 1 [file pharmaceuticals-14-01098-s001.zip › pharmaceuticals-1414350-supplementary.pdf]

# Supporting Information

# HPLC/ESI-MS and NMR analysis of chemical constitutes in bioactive extract from the root nodule of *Vaccinium emarginatum*

**Hsiang-Ming Huang [1†], Chien-Yi Ho [2,3†], Geng-Ruei Chang [4], Wei-Yau Shia [5], Cheng-Hung Lai [5], Chih-Hao Chao [6,*] and Chao-Min Wang [4,*]**

[1] Neurosurgery Department, China Medical University Hsinchu Hospital, Hsinchu 302, Taiwan; d10190168@gmail.com (H.-M.H.)
[2] Department of Biomedical Imaging and Radiological Science, China Medical University, Taichung 404, Taiwan; samsam172@yahoo.com.tw (C.-Y.H.)
[3] Division of Family Medicine, Physical Examination Center, and Department of Medical Research, China Medical University Hsinchu Hospital, Hsinchu 302, Taiwan; samsam172@yahoo.com.tw (C.-Y.H.)
[4] Department of Veterinary Medicine, National Chiayi University, 580 Xinmin Road, Chiayi 600023, Taiwan; grchang@mail.ncyu.edu.tw (G.-R.C.)
[5] Department of Veterinary Medicine, National Chung-Hsing University, 145 XingDa Road, Taichung 402, Taiwan; vmwyshia@nchu.edu.tw (W.-Y.S.); chlai@dragon.nchu.edu.tw (C.-H.L.)
[6] Division of chest medicine, Attending physician of Chang Bing Show Chwan Memorial Hospital, 6 Lugong Road, Lukang Township, Changhua 505029, Taiwan
[†] These authors contributed equally to this work
[*] Correspondence: leowang@mail.ncyu.edu.tw (C.-M.W.); drchao028@gmail.com (C.-H.C)
Tel.: +886-5-2732970 (C.-M.W.); +886- 975-617918 (C.-H.C)

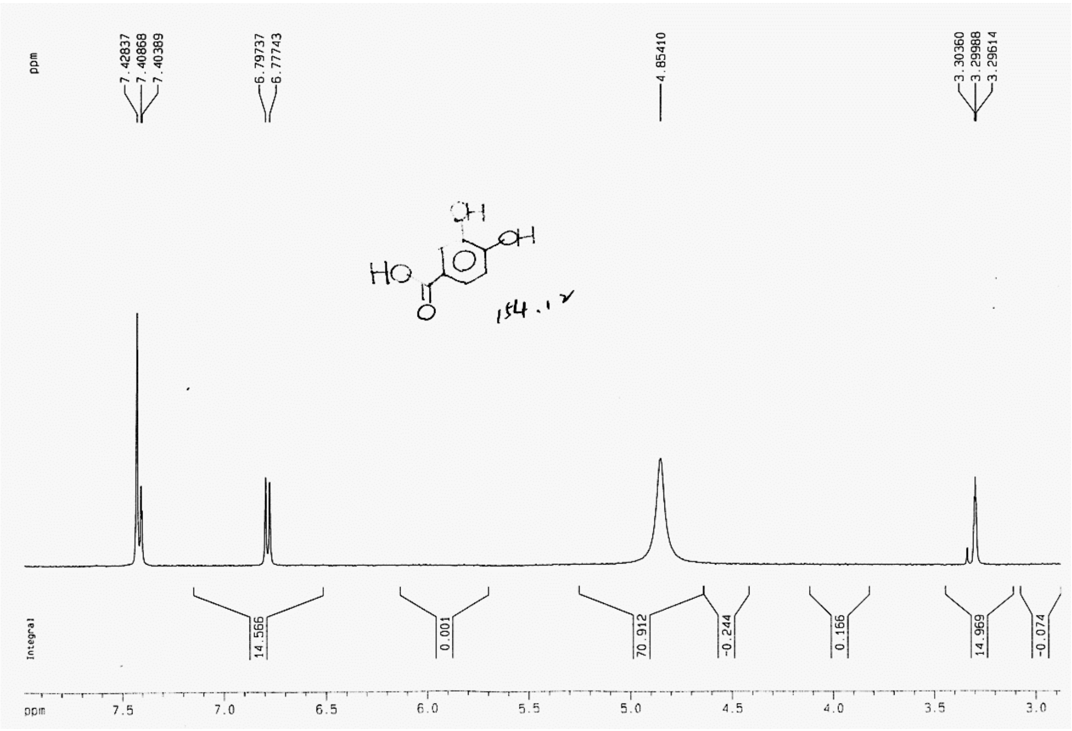


Figure S1. $^{1}$H NMR spectrum of compound **1**.

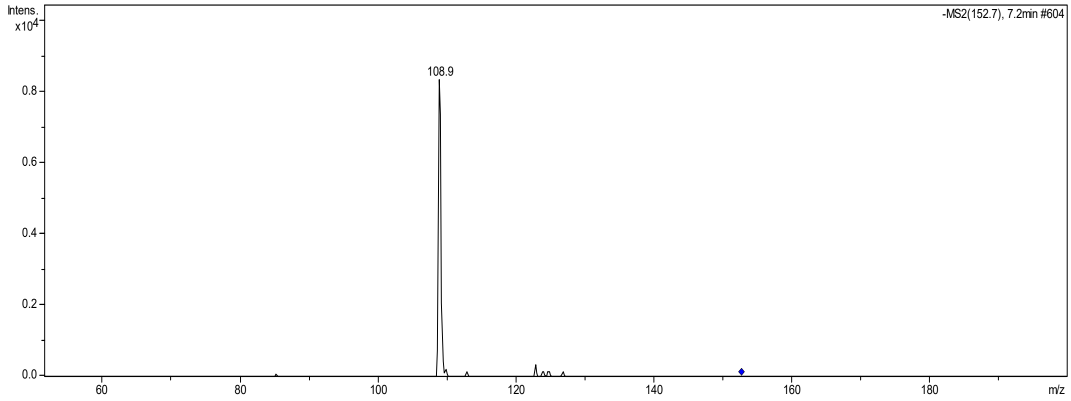


Figure S2. MS/MS spectrum of compound **1**.

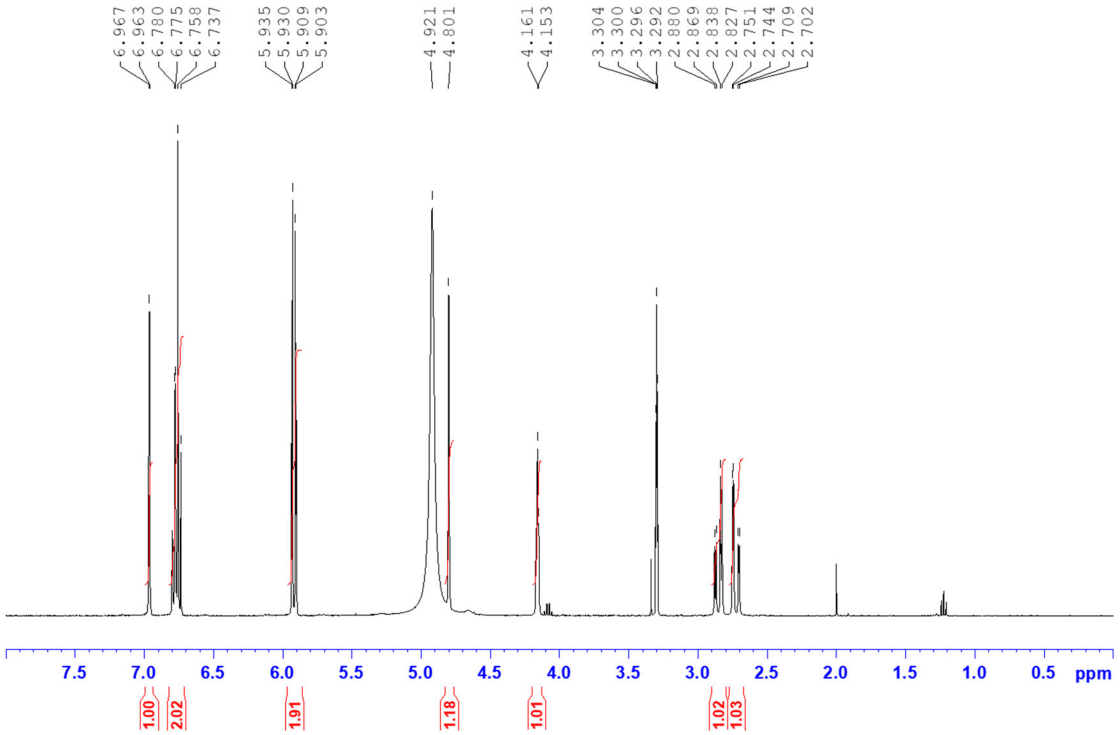


Figure S3. $^{1}$H NMR spectrum of compound **2**.

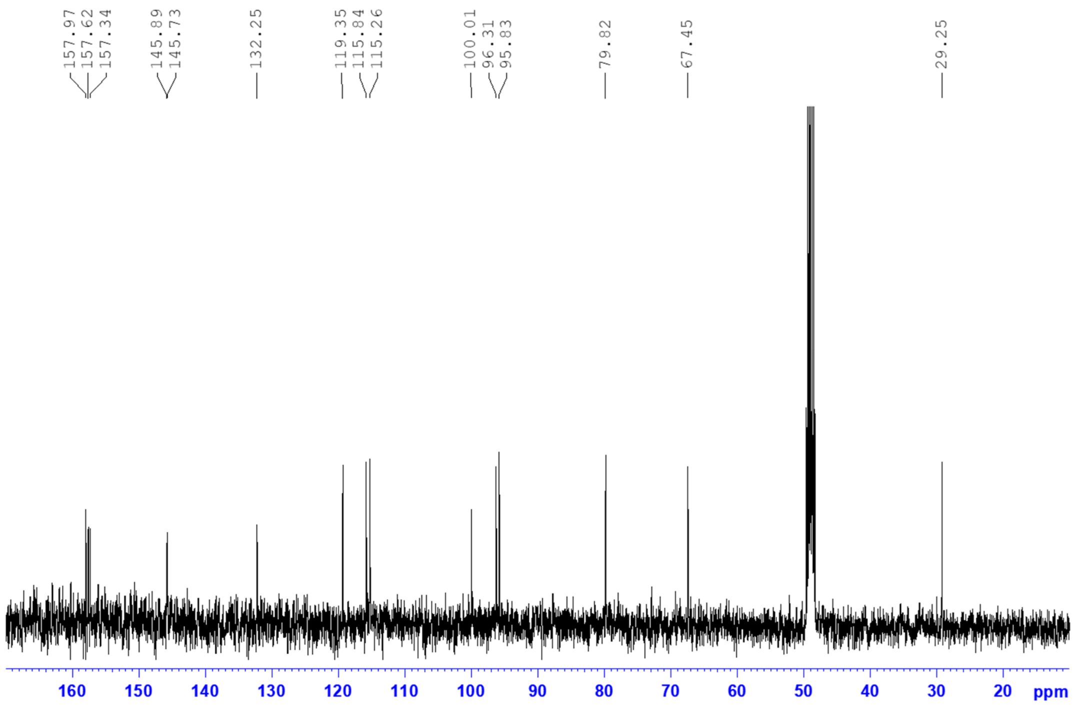


Figure S4. $^{13}$C NMR spectrum of compound **2**.

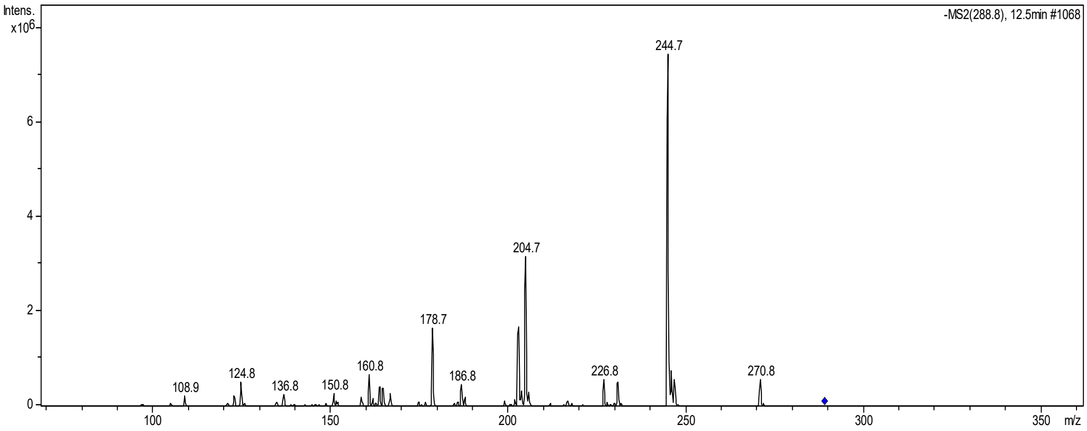


Figure S5. MS/MS spectrum of compound **2**.

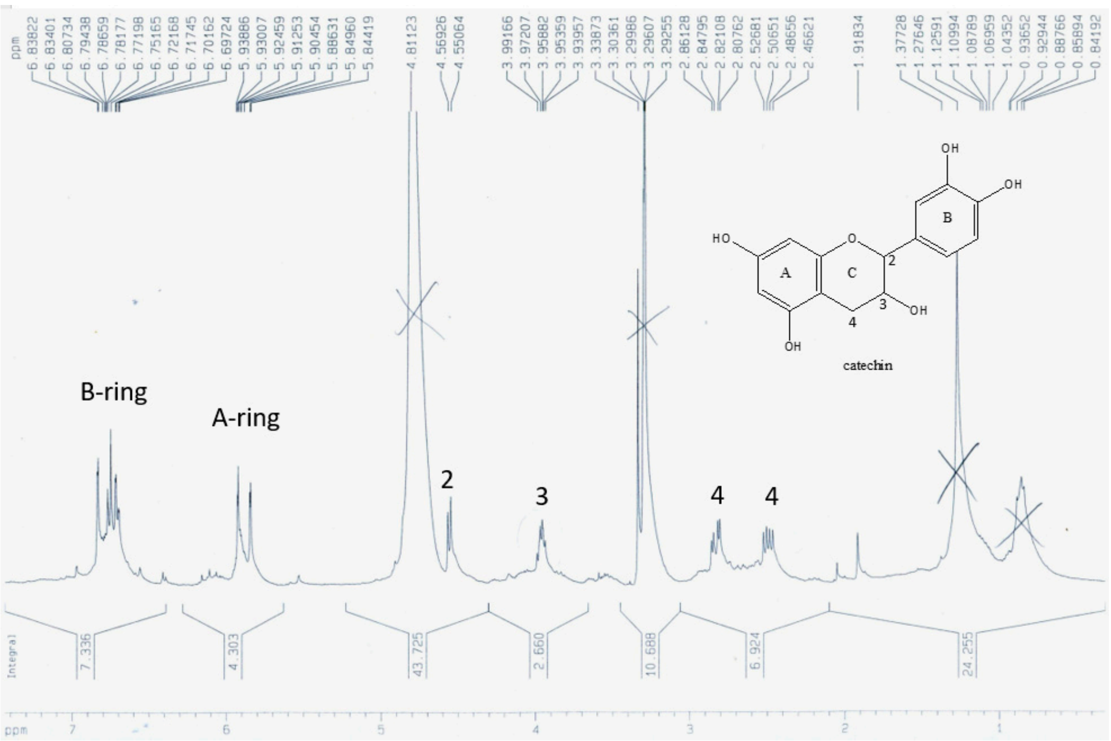


Figure S6. $^{1}$H NMR spectrum of compound **3**.

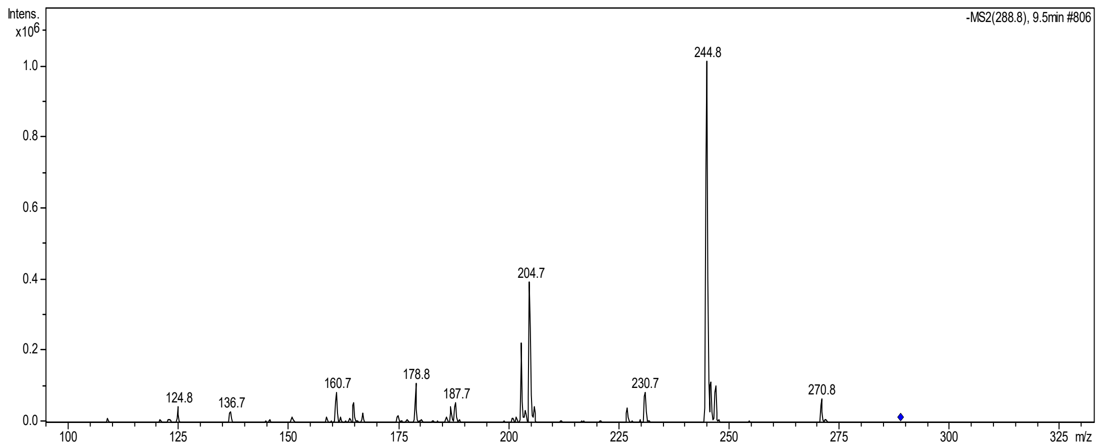


Figure S7. MS/MS spectrum of compound **3**.

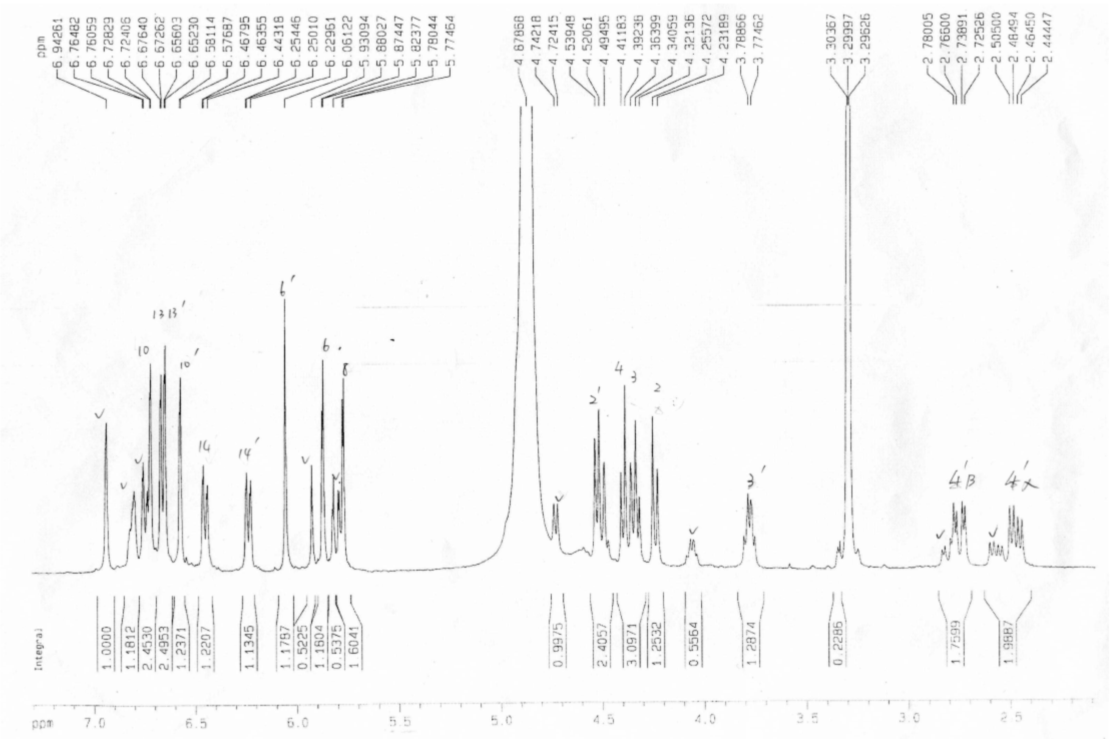


Figure S8. ¹H NMR spectrum of compound **4**.

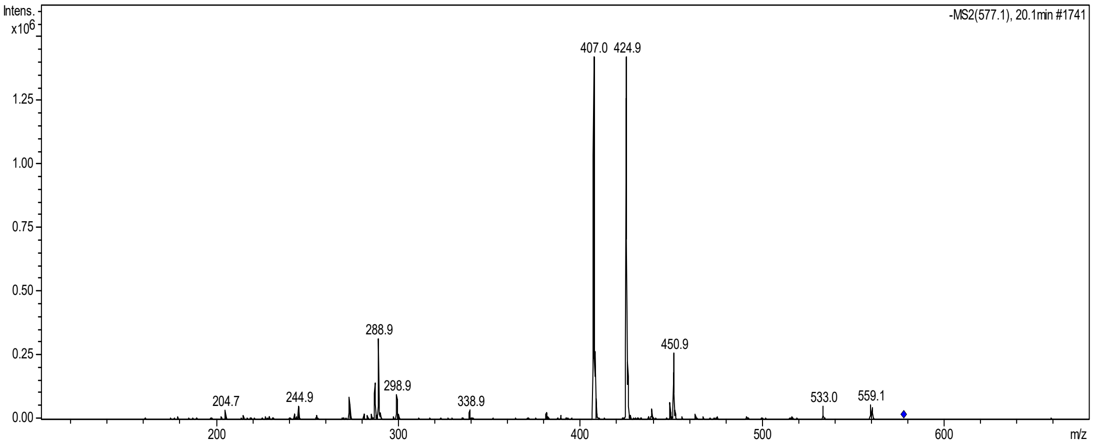


Figure S9. MS/MS spectrum of compound **4**.

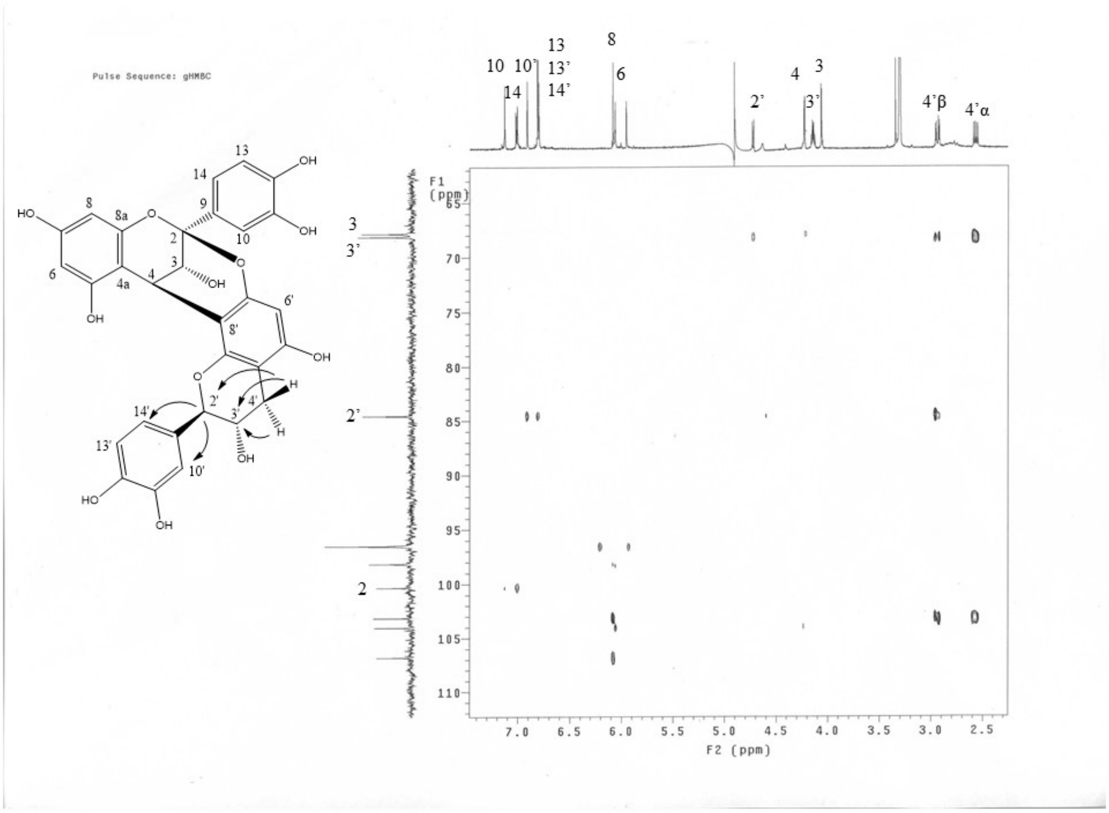


Figure S10. HMBC spectrum of compound **5**.

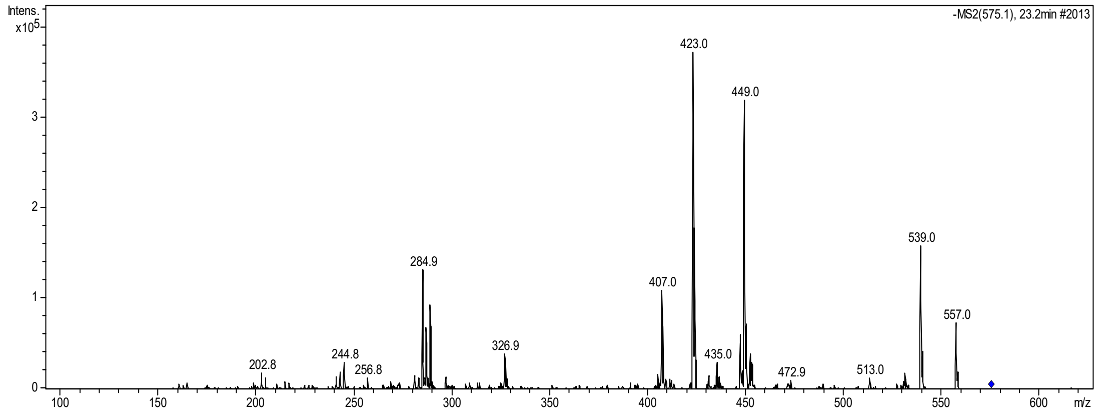


Figure S11. MS/MS spectrum of compound **5**.

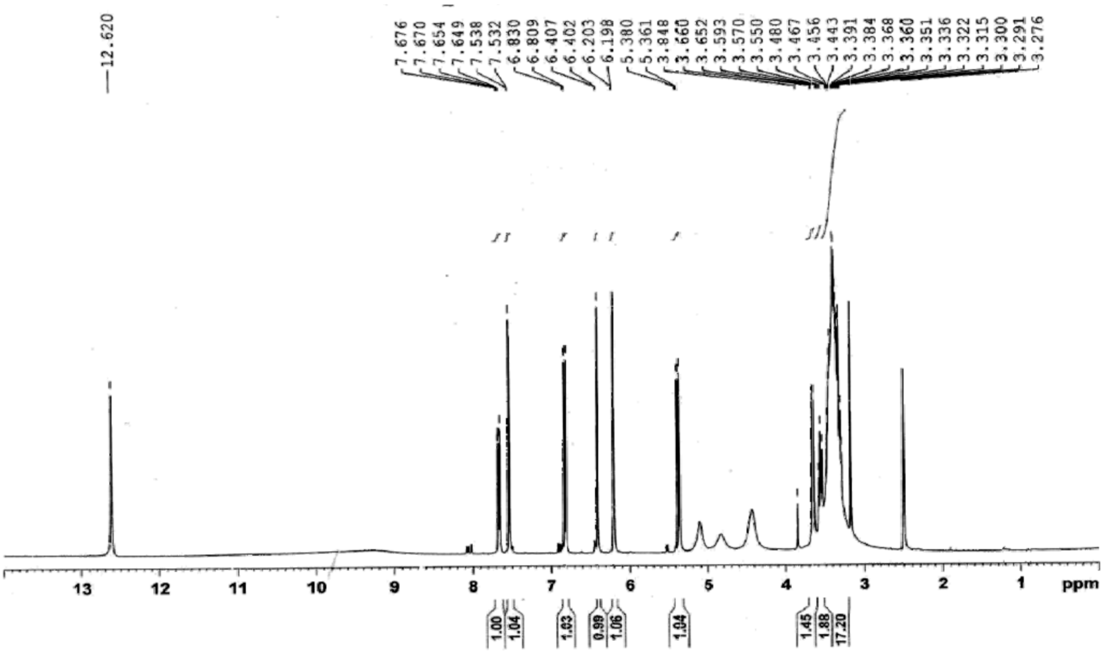


Figure S12. $^{1}H$ NMR spectrum of compound **6**.

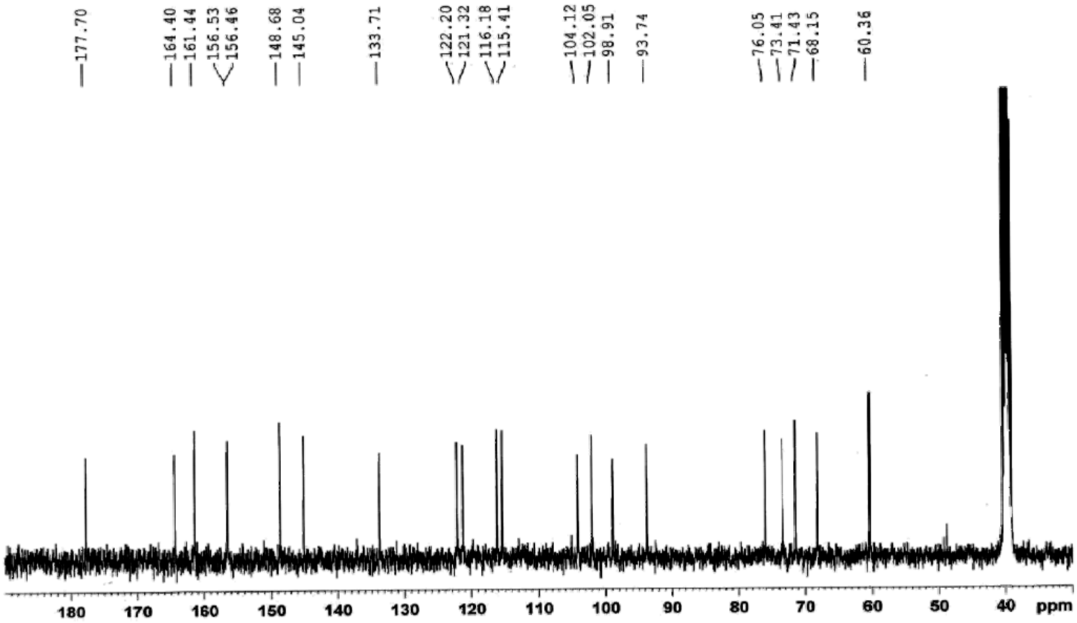


Figure S13. $^{13}C$ NMR spectrum of compound **6**.

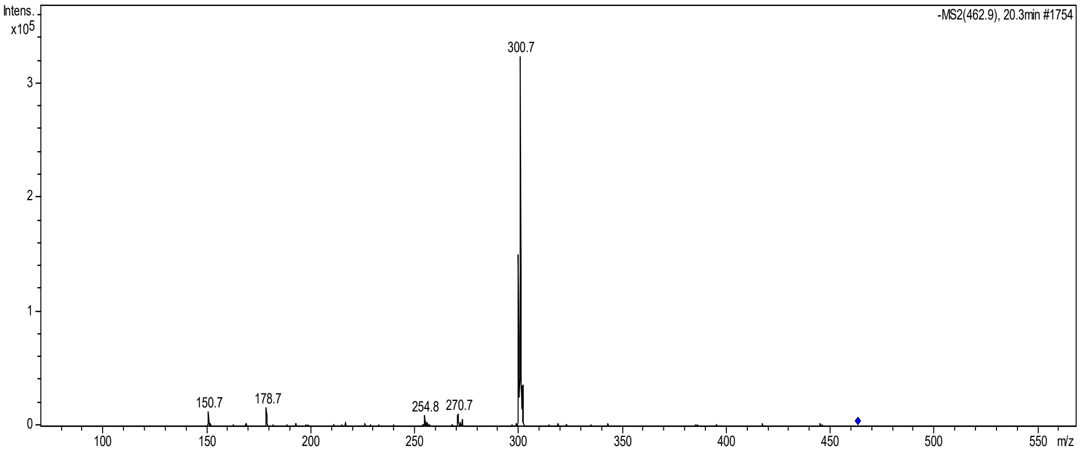


Figure S14. MS/MS spectrum of compound **6**.

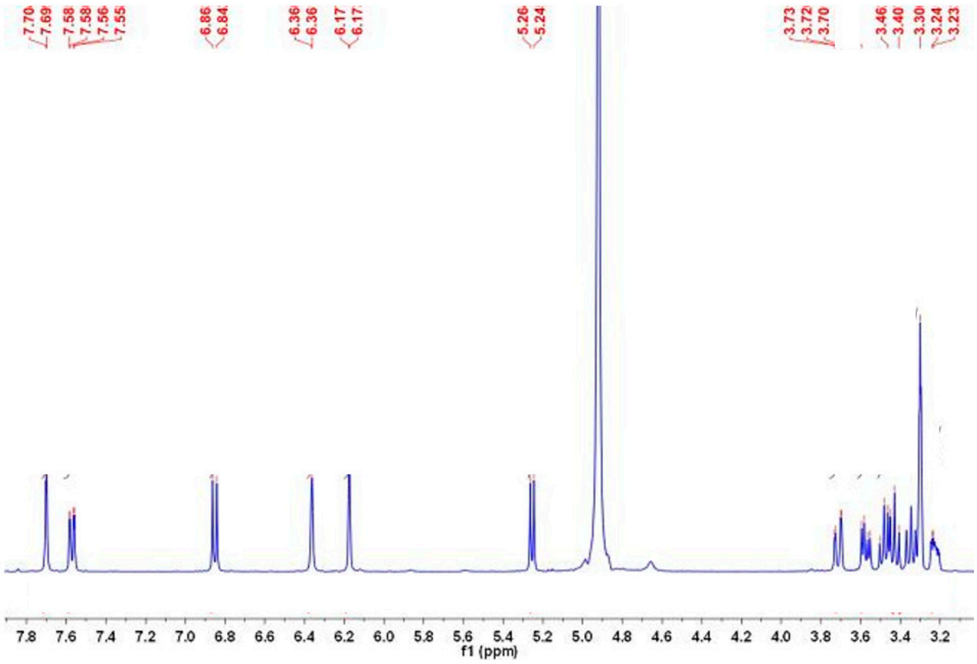


Figure S15. $^{1}$H NMR spectrum of compound **7**.

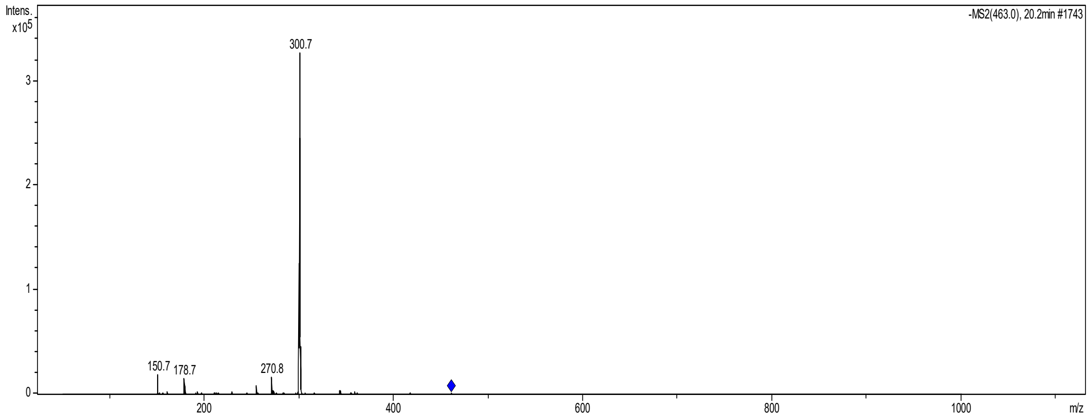


Figure S16. MS/MS spectrum of compound **7**.

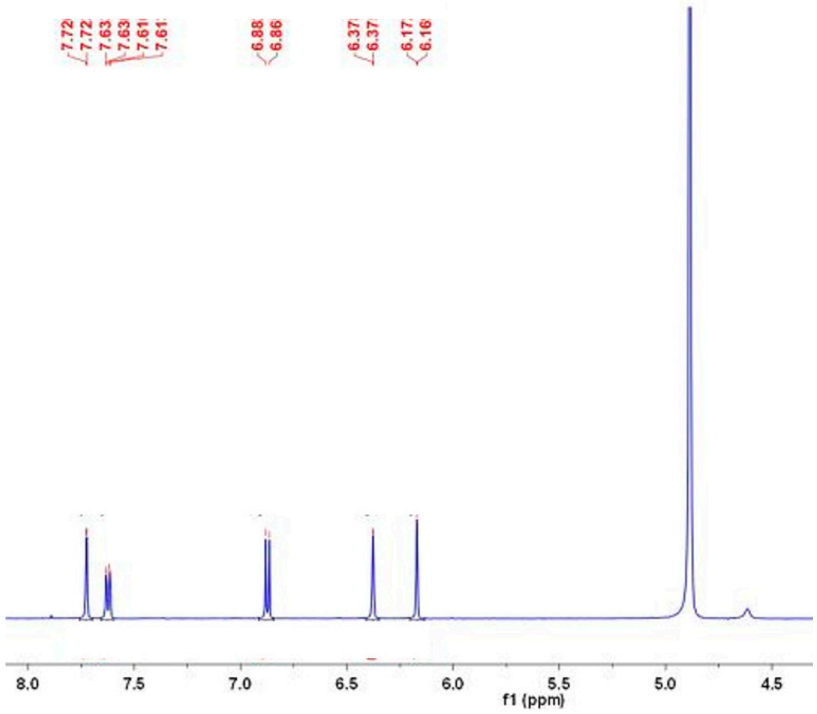


Figure S17. $^1$H NMR spectrum of compound **8**.

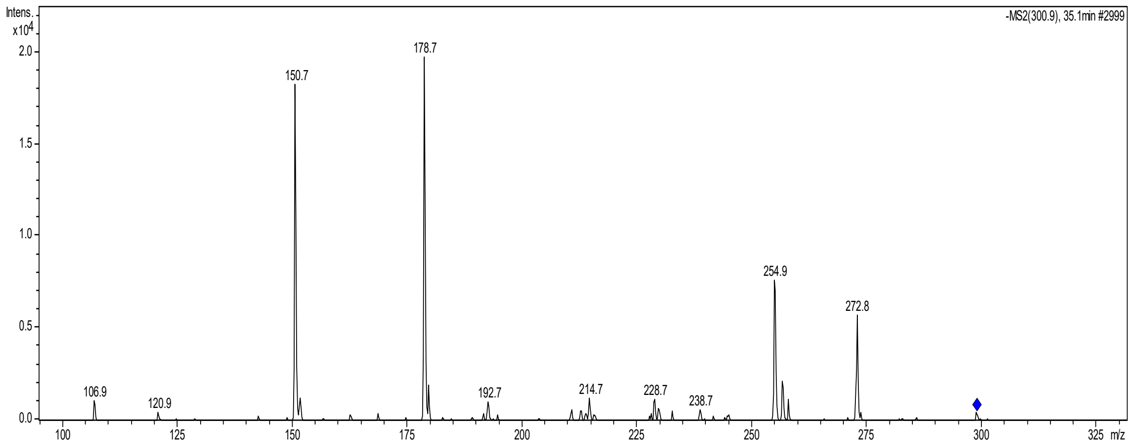


Figure S18. MS/MS spectrum of compound **8**.

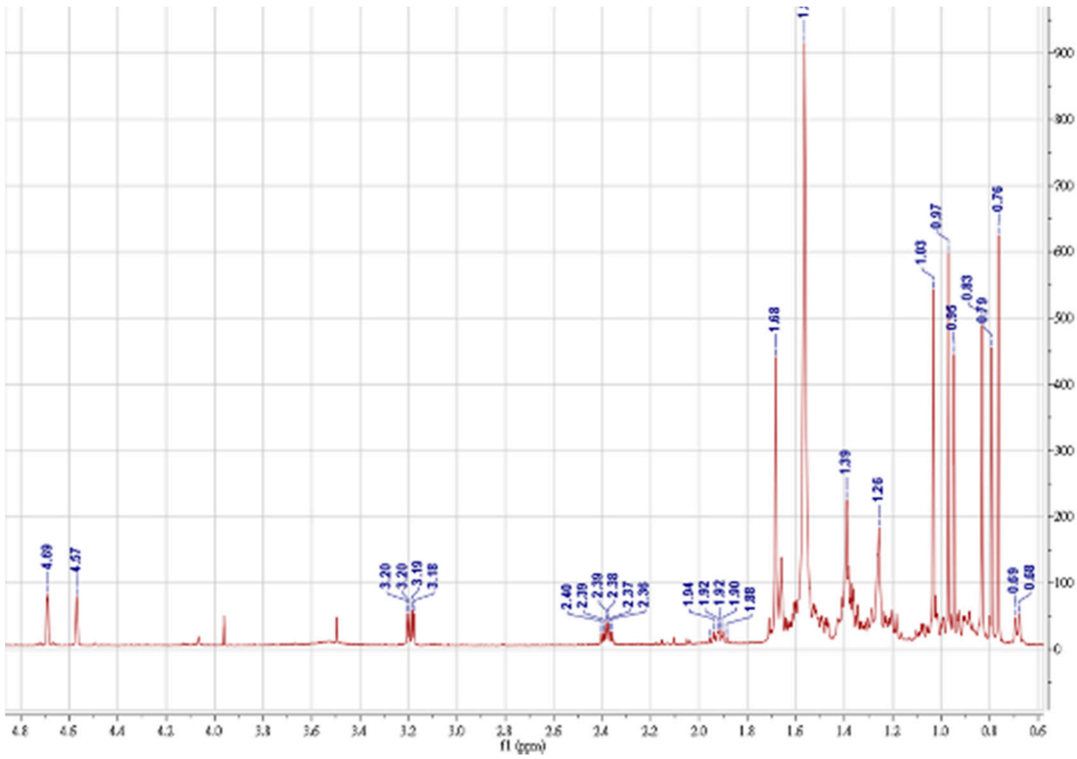


Figure S19. $^{1}$H NMR spectrum of compound **9**.

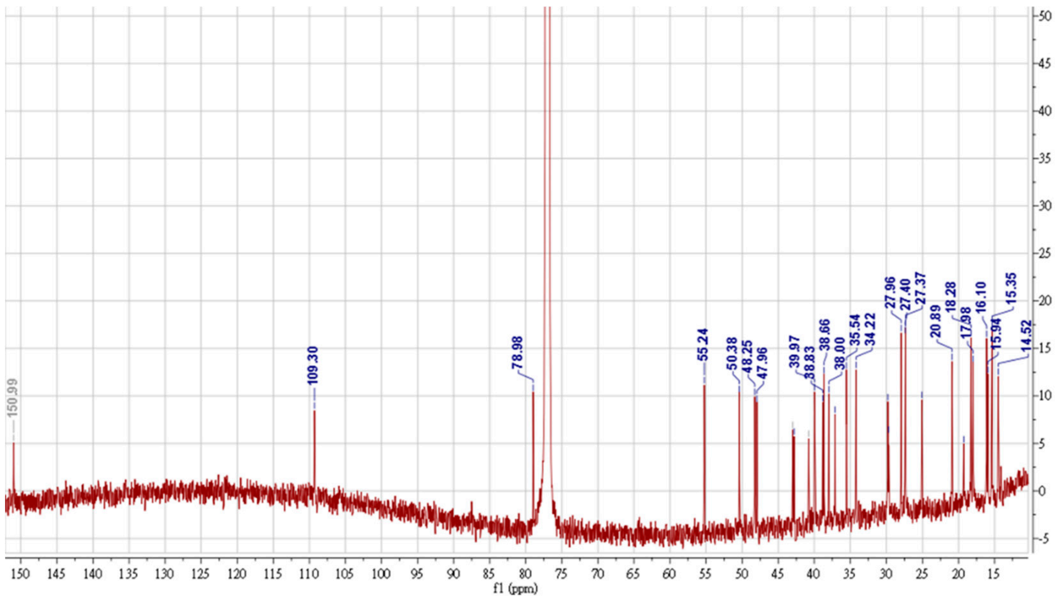


Figure S20. $^{13}$C NMR spectrum of compound **9**.

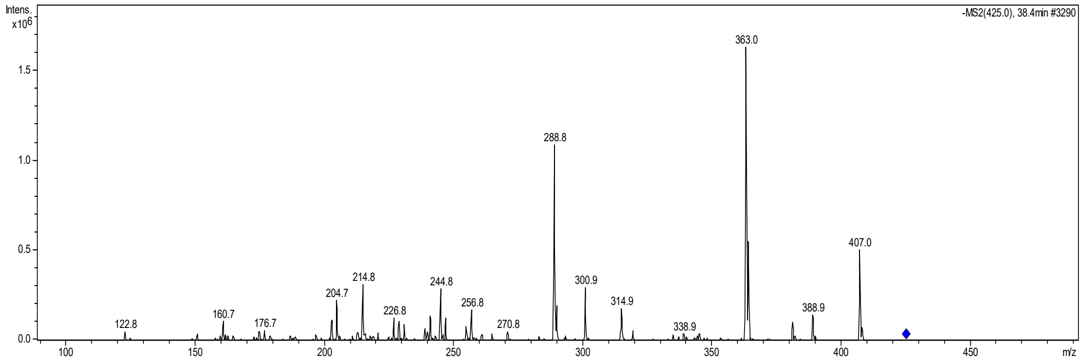


Figure S21. MS/MS spectrum of compound **9**.

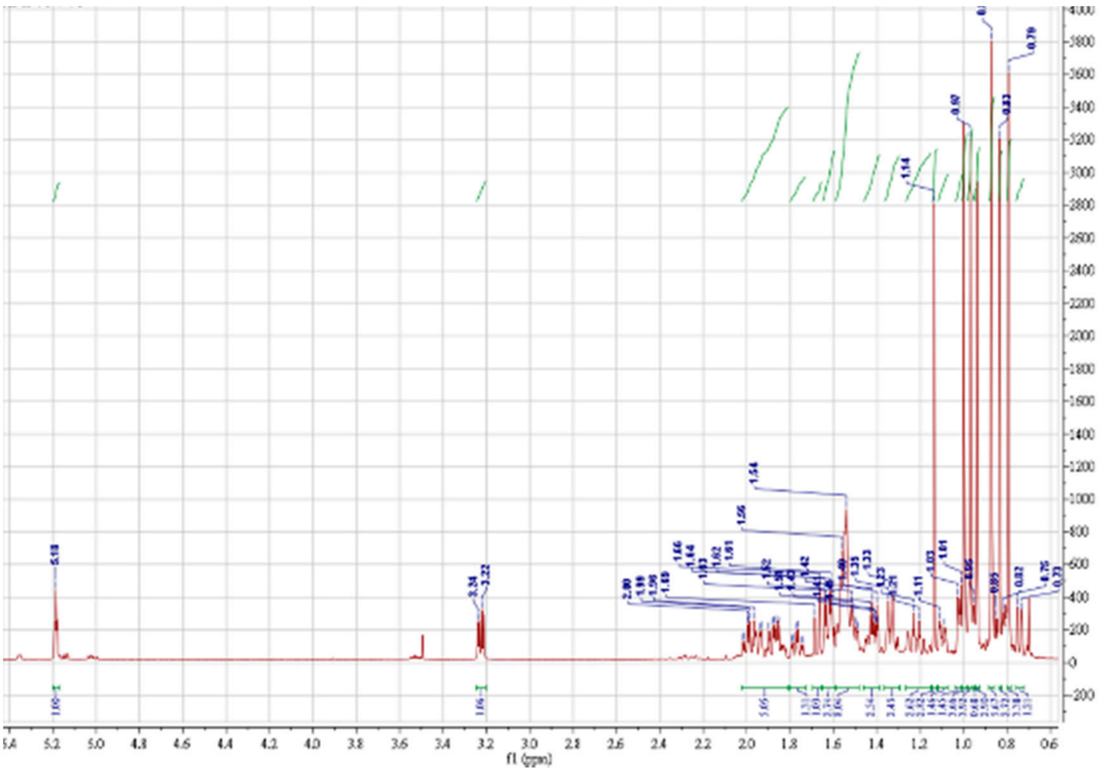


Figure S22. $^{1}$H NMR spectrum of compound **10**.

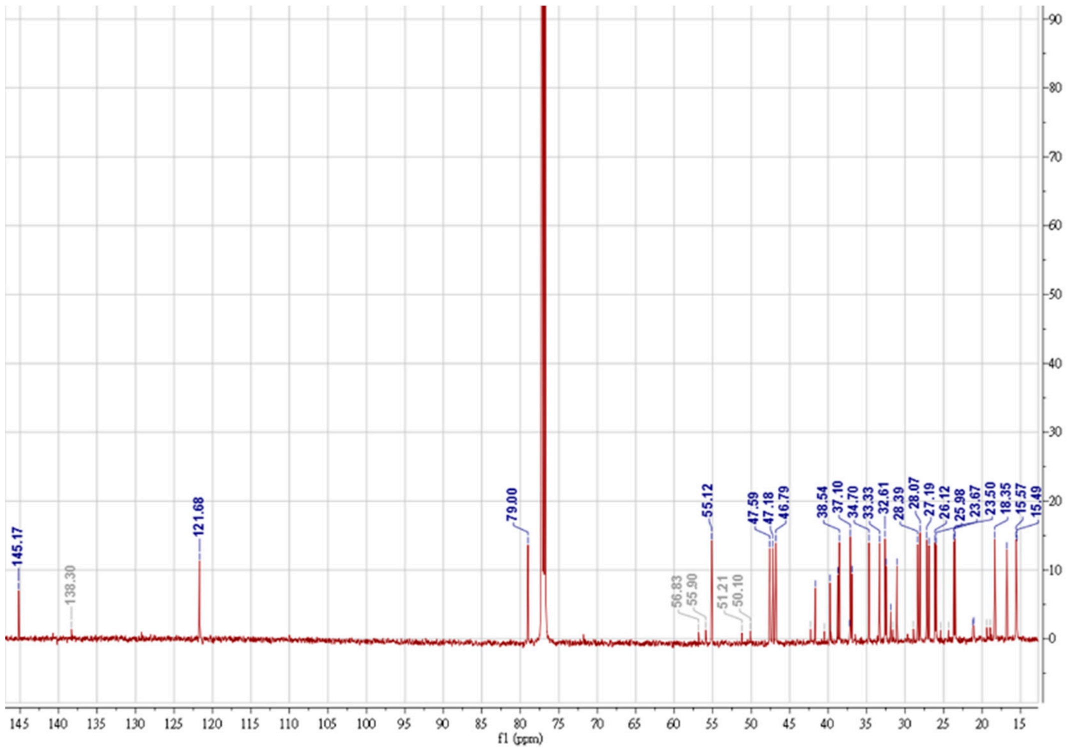

Figure S23. $^{13}$C NMR spectrum of compound **10**.

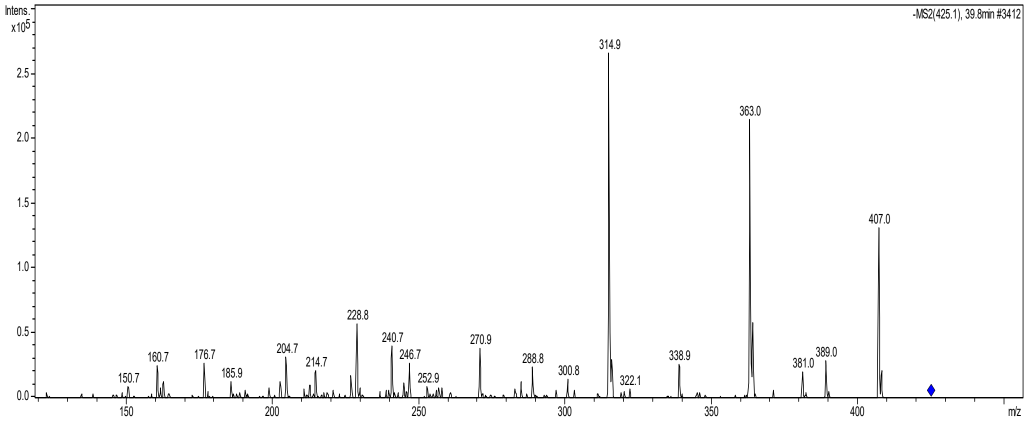

Figure S24. MS/MS spectrum of compound **10**.

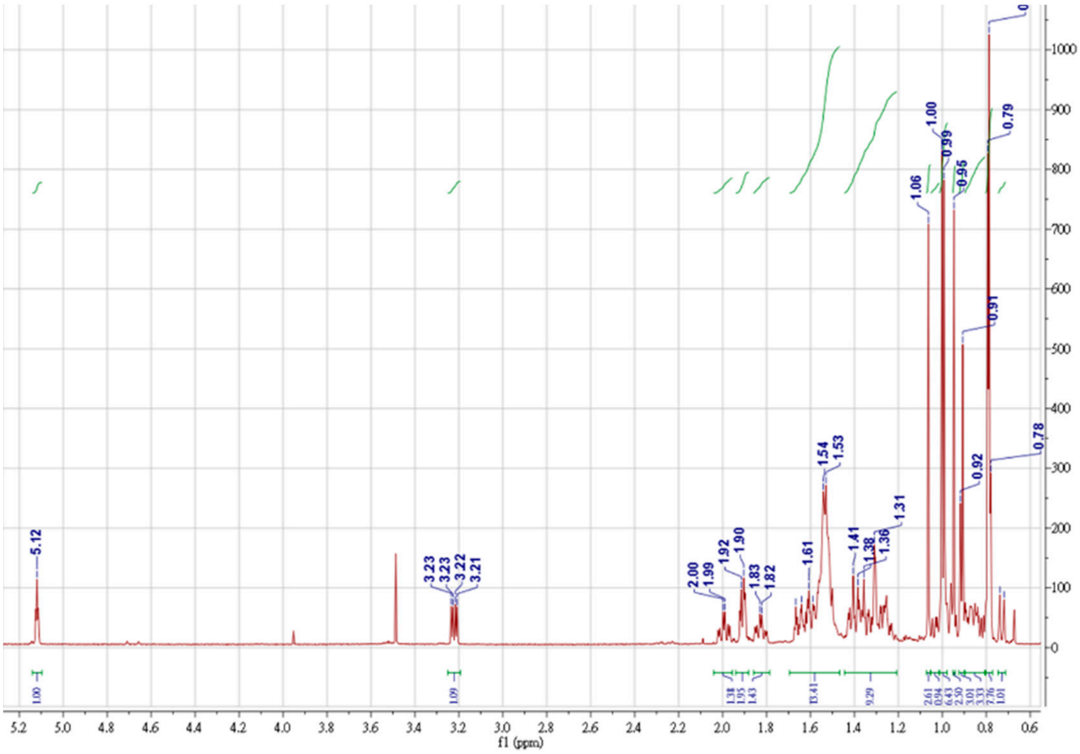


Figure S25. $^{1}$H NMR spectrum of compound **11**.

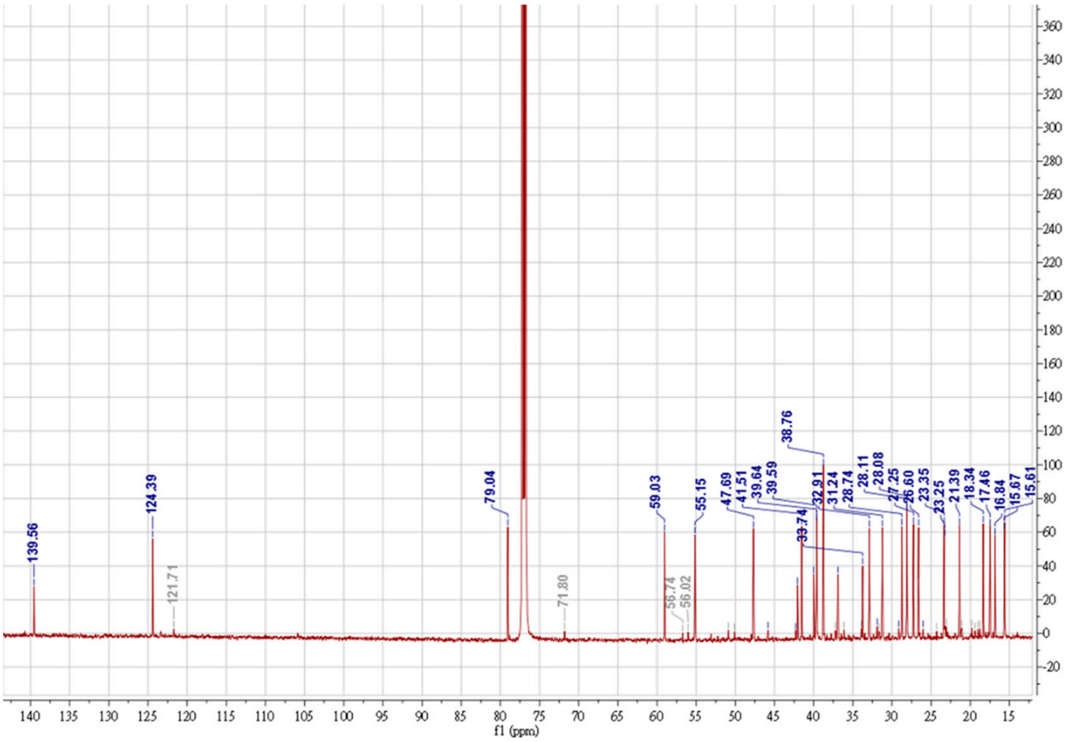


Figure S26. $^{13}$C NMR spectrum of compound **11**.

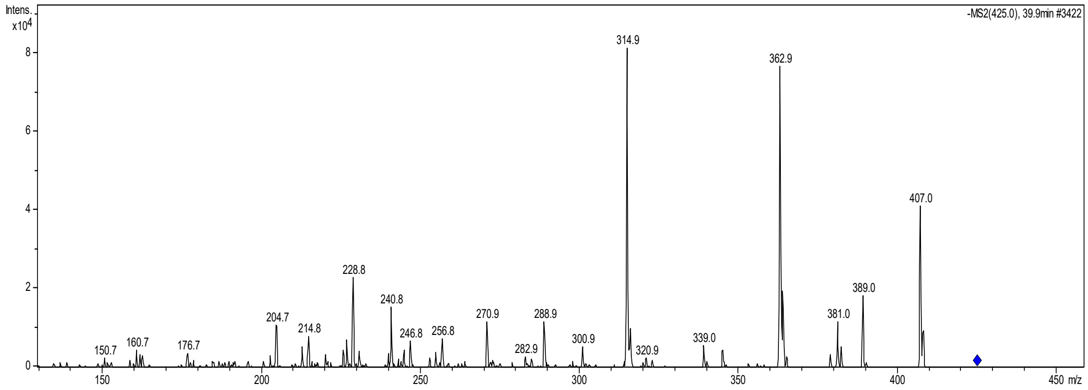


Figure S27. MS/MS spectrum of compound **11**.
